# Investigating the Mechanistic Link Between Lactate-Induced Histone Lactylation and Cellular Senescence in Osteoarthritis Chondrocytes: Implications for Therapy

**DOI:** 10.7150/ijbs.126483

**Published:** 2026-02-18

**Authors:** Rongtai Zuo, Chenliang Wu, Zhiqi Lin, Lingchi Kong, Feng Wang, Xuancheng Zhang, Jianxing Wei, Yi Lin, Erpeng Yang, Qinglin Kang, Shichang Zhao, Junjie Guan, Jinzhong Zhao

**Affiliations:** 1 Department of Sports Medicine, Shanghai Sixth People's Hospital Affiliated to Shanghai Jiao Tong University School of Medicine, Shanghai 200233, China.; 2 Department of Orthopedics, Shanghai Sixth People's Hospital Affiliated to Shanghai Jiao Tong University School of Medicine, Shanghai 200233, China.; 3 Department of Orthopedics, Fuzhou Second General Hospital, Fujian 350007, China.

**Keywords:** osteoarthritis, lactate, chondrocyte, senescence, lactylation, H4K12la

## Abstract

Chondrocyte senescence accelerates osteoarthritis (OA) progression, with dysregulated glycolysis potentially driving this process through lactate accumulation and histone lactylation. However, the mechanistic connection between lactate-induced lactylation and cellular senescence in OA chondrocytes remains poorly understood. This study elucidates this relationship and explores its therapeutic relevance. Using clinical OA cartilage, animal models, and IL-1β-stimulated chondrocytes, we demonstrated enhanced glycolysis and senescence activities. Increased glycolytic activity and lactate accumulation promoted cellular senescence in the OA microenvironment. Elevated lactate levels increased global lactylation, with H4K12la as the predominant mark. H4K12la-targeted CUT&TAG analysis identified TRIM29 as a key regulator. H4K12la promotes TRIM29 transcription, which activates the PI3K-AKT pathway via direct and EGFR-mediated mechanisms, leading to autophagy inhibition and senescence. Interventions such as SIRT1 overexpression or intra-articular oxamate injection reduced lactylation, inhibited glycolysis, and mitigated cartilage degeneration. These findings demonstrate that glycolytic lactate-induced H4K12la fosters senescence through TRIM29-mediated PI3K-AKT activation, highlighting the inhibition of glycolysis or lactylation as a promising therapeutic strategy for OA.

## 1. Introduction

Osteoarthritis (OA), a prevalent and debilitating degenerative joint disease, imposes a substantial and escalating global health burden, currently affecting over 500 million individuals worldwide^1^. This condition drives significant healthcare costs and morbidity. Progressive loss of articular cartilage, accompanied by subsequent involvement of the synovium, ligaments, and muscles, leads to characteristic pain and functional deficits^2^. Consequently, elucidating the fundamental pathological mechanisms of OA is paramount for developing effective disease-modifying therapies within the precision medicine paradigm.

Cellular senescence represents a pivotal driver of OA pathogenesis. Senescent chondrocytes play a key role in joint degeneration; these permanently arrested cells exhibit a damaging SASP, secreting factors such as proinflammatory cytokines (IL-1β, IL-6 and IL-10) and matrix-degrading enzymes (MMP3, MMP13) into the local microenvironment^3^, ^4^. This secretory profile not only diminishes chondrocyte proliferative capacity, impairing cartilage repair but also accelerates cartilage catabolism and fuels chronic intra-articular inflammation^5^, ^6^. Selectively eliminating senescent chondrocytes has thus emerged as a promising therapeutic avenue^7^. However, the intrinsic mechanisms governing chondrocyte senescence in OA remain incompletely understood, hindering targeted intervention development.

Growing evidence implicates chondrocyte energy metabolism dysregulation as critical to OA progression^8^-^11^. Unlike most tissues, articular cartilage exists under physiological hypoxia, rendering chondrocytes uniquely sensitive to metabolic shifts^12^. Healthy chondrocytes utilize both oxidative phosphorylation and aerobic glycolysis for ATP production. However, OA progression coincides with a profound metabolic shift in chondrocytes, characterized by markedly increased aerobic glycolysis. This metabolic reprogramming drives significant intra-articular lactate accumulation, which inhibits chondrocyte anabolic activity and accelerates articular cartilage degradation^13^. More importantly, lactate produced by glycolysis has a mutually reinforcing relationship with chronic inflammation, which establishes a pathological positive-forward loop^13^, ^14^. Consequently, glycolysis-derived lactate exacerbates intra-articular microenvironment instability, thereby potentiating OA pathogenesis.

The interplay between glycolysis and cellular senescence is highly context dependent. Elevated expression of glycolytic enzymes has been reported in some senescent cell populations^15^, and pharmacologic enolase 1 inhibition, which suppresses glycolysis, mitigates UVA-induced senescence^16^. Conversely, other studies reported diminished glycolytic flux in senescent cells, with hypoxic activation of glycolysis slowing senescence progression^17^. Moreover, extracellular vesicle-derived lncRNA VIM-AS1 promotes glycolysis, which consequently alleviates cellular senescence and facilitates diabetic wound healing^18^. This evidence suggests that divergent regulatory relationships exist between glycolytic activity and senescence, contingent upon the specific pathological conditions involved. Crucially, the nature and mechanistic basis of this relationship within the OA chondrocyte environment remain entirely unexplored.

Lactylation, a recently identified histone mark induced by lactate as a substrate^19^, modulates gene expression and has since been documented in histone and nonhistone proteins. It influences diverse biological processes, including cellular signaling, proteostasis, and epigenetic regulation, across multiple pathologies^20^-^23^. In OA, FSTL1 has been shown to enhance glycolysis, thereby driving H3K18 lactylation to induce cartilage fibrosis and damage^24^. Conversely, targeted delivery of lysyl oxidases via nanomedicine can modulate histone lactylation to promote cartilage repair^25^. These studies underscore the critical role of lactylation in OA cartilage damage. However, the underlying mechanisms by which lactylation regulates chondrocyte senescence remain elusive.

This study aims to elucidate glycolytic remodeling in OA and reveal the mechanistic link between excess glycolysis-derived lactate and senescence in articular chondrocytes. Our findings provide the first evidence of reciprocal glycolysis‒senescence regulation in chondrocytes and reveal that lactylation is a novel pathogenic mechanism and therapeutic target in OA.

## 2. Materials and Methods

### 2.1 Human knee cartilage sample collection

All procedures involving human participants Table S1 were approved by the Ethics Committee of Shanghai Sixth People's Hospital and conducted in accordance with the Declaration of Helsinki (Approval No. 2023-KY-026(K)). Informed consent in writing was secured from all participants before they were included in the study. Healthy knee cartilage samples were obtained from individuals who underwent anterior cruciate ligament reconstruction (ACLR) surgery, whereas osteoarthritic cartilage samples were obtained from patients diagnosed with OA who were receiving total knee arthroplasty (TKA).

### 2.2 Cartilage transcriptome sequencing

Total RNA was extracted from cartilage samples using TRIzol® Reagent (Invitrogen) and assessed for quality with a NanoDrop ND-2000 spectrophotometer. Only high-quality RNA samples (OD260/280 = 1.8-2.2; OD260/230 ≥ 2.0; RQN ≥ 6.5; 28S:18S ≥ 1.0; total RNA > 1 μg) were used for subsequent steps. Library preparation and sequencing were performed by Shanghai Majorbio Biopharm Biotechnology Co., Ltd. using the Illumina® Stranded mRNA Prep Ligation Kit and the NovaSeq X Plus platform (PE150). Raw reads were processed with fastp for adapter trimming and quality control. Clean reads were aligned to the reference genome using HISAT2, and transcripts were assembled with StringTie. Gene expression was quantified as transcripts per million (TPM) via RSEM. Differential expression analysis was conducted with DESeq2, considering genes with |log₂FC| ≥ 1 and FDR < 0.05 as significant. Functional enrichment analysis for GO and KEGG terms was performed using a hypergeometric test (P < 0.05).

### 2.3 Establishment of animal OA model

All animal experiments were approved by the Animal Welfare and Ethical Committee of Shanghai Pudong Hospital (Approval No. 20241216-001). An OA model was established via anterior cruciate ligament transaction (ACLT) in 2-month-old male Sprague-Dawley (SD) rats as previously described^26^. Briefly, under anesthesia, the right knee joint was exposed via capsulotomy, and the anterior cruciate ligament was transected to induce joint instability. Sham-operated control animals underwent capsulotomy without ligament transaction. After 4 weeks, the specific intervention method was conducted every week. The rats were divided into four group: sham group, OA group, OA + SRT1720 group, and OA + OXA group. All animals were euthanized at 8 weeks post-surgery, and the knee joints were harvested for subsequent analysis.

### 2.4 Isolation and culture of primary chondrocytes

The primary chondrocytes used for in vitro experiments were isolated from 2-week-old SD rats. Following euthanasia, the rats were disinfected with 75% ethanol for 15 minutes and then transferred to a sterile surgical field. The knee joints were dissected, the surrounding muscle tissue was carefully removed, and the joints were placed in sterile phosphate-buffered saline (PBS) containing 1% penicillin‒streptomycin (P-S, ScienCell, Wuxi, China). Under a laminar flow hood, cartilage tissues from the tibial plateau and distal femur were carefully dissected and transferred to a culture dish. The collected cartilage was minced into approximately 2 mm³ fragments and digested with 2.5% trypsin-EDTA for 30 minutes at 37°C. Following the aspiration of the trypsin solution, the fragments underwent an additional digestion with collagenase II (Sigma) at a concentration of 2 mg/ml for 4 hours at 37°C with gentle agitation. The resulting cell suspension was passed through a 70 μm filter and then centrifuged to obtain a cell pellet. This pellet was then resuspended in DMEM/F12 medium (Gibco, Carlsbad, USA), which was supplemented with 10% fetal bovine serum (FBS; Gibco, South American origin) and 1% P-S antibiotics, before being transferred into culture flasks. The medium was changed after 3 days. By day 7, the primary chondrocytes had formed distinct clusters and reached sufficient confluence. The cells were then trypsinized, passaged, and expanded for subsequent experimental use.

### 2.5 Synthesis and transfection of siRNAs and plasmids

siRNAs targeting TRIM29 and SIRT1, along with overexpression plasmids for TRIM29 and SIRT1, were designed and synthesized by Zorin Biotechnology Co., Ltd. (Shanghai, China). The siRNA sequences used were as follows: siRNA-TRIM29: KD1: sense 5′-GGAGUCAGUAGACGACAAATT-3′, antisense 5′-UUUGUCGUCUACUGACUCCTT-3′; KD2: sense 5′-GACCGGAUCAAGAGCUUUATT-3′, antisense 5′-UAAAGCUCUUGAUCCGGU CTT-3′; KD3: sense 5′-GUGCGUUGAUGAGCAAUUATT-3′, antisense 5′-UAAUUGC UCAUCAACGCACTT-3′. siRNA-SIRT1: KD1: sense 5′-CUUCGAAAUUAUACUCAAATT -3′, antisense 5′-UUUGAGUAUAAUUUCGAAGTT -3′; KD2: sense 5′- GCAAAGGAGCAGA UUAGUATT-3′, antisense 5′-UACUAAUCUGCUCCUUUGCTT-3′; KD3: sense 5′-GAACAA AGUUGACGAUUUATT-3′, antisense 5′-UAAAUCGUCAACUUUGUUCTT-3′. Plasmids encoding TRIM29 and SIRT1 were constructed with the following flanking sequences: TRIM29 plasmid: forward: 5′-ttcggatccaccATGgaaggtgccgatgcc-3′; reverse: 5′-tccaatgaggcacccctcgagg gaggtgga-3′; SIRT1 plasmid: forward: 5′- ttcggatccaccATGtgcctgtgcagtgga-3′; reverse: 5′- aaatcagagcactatctcgagggaggtgga-3′.

For the transfection process, the manufacturer's instructions were followed using Lipofectamine 3000 (Thermo Fisher Scientific, L3000001). SiRNA oligos or plasmids were first diluted in Opti-MEM (Thermo Fisher Scientific, 31985070), subsequently combined with the Lipofectamine 3000 reagent, and allowed to incubate at RT for 20 minutes. Next, the transfection mixture was introduced into chondrocytes that had been cultured in 24-well plates and cocultured for a duration of 6 hours. Later, the medium was exchanged for complete growth medium. The cells were then maintained for an additional 48 hours prior to further experiments aimed at evaluating the transfection efficiency.

### 2.6 Quantitative reverse transcription polymerase chain reaction (qRT-PCR)

Gene expression levels of target genes were quantified via qRT‒PCR. Total RNA was extracted via an EZ-press RNA Purification Kit (EZ Bioscience, Suzhou, China, B0004D) according to the manufacturer's instructions. The RNA concentration and purity were assessed via a NanoDrop microspectrophotometer; samples with OD260/OD280 ratios between 1.7 and 2.0 were deemed suitable for further analysis. cDNA was synthesized from total RNA via the Color All-in-one Reverse Transcription Kit (with DNase) (EZ Bioscience, RT3C). qPCR was performed with 2× Color SYBR Green qPCR Master Mix (ROX 2) (EZ Bioscience, A0012R2). GAPDH was used as an endogenous reference gene for normalization. All sequences of primers used in this study are provided in supplementary Table S2.

### 2.7 Western blotting

The levels of protein expression in articular cartilage tissues and cultured cells were assessed through western blot analysis. Cartilage samples (20 mg) or pretreated cells derived from six-well plates were lysed with 200 μL of RIPA buffer (Epizyme, Shanghai, China, PC101) supplemented with 1% PMSF (Epizyme, GRF101) on ice for 30 minutes. The lysates were subjected to centrifugation at 12,000 × g for 15 minutes at 4°C to obtain the total protein supernatant. And the histone in cell nucleus was obtained via Histone Extraction Kit (Proteintech, Wuhan, China, PK10022). Protein concentrations were measured with a BCA protein assay kit (Epizyme, ZJ102). Equal protein quantities were separated via 10-15% SDS‒PAGE gels (Epizyme) and subsequently transferred to PVDF membranes (Millipore, Billerica, MA, USA, 24937-79-9). The membranes were blocked with protein-free rapid blocking buffer (Epizyme, PS108) for 15 minutes at room temperature (RT) before being incubated with primary antibodies at 4°C overnight. Following washing, the membranes were treated with species-matched HRP-conjugated secondary antibodies for 2 hours at RT. The protein bands were detected via the Omni-ECL™ Femto Light Chemiluminescence Kit (Epizyme, SQ201) and quantified via Quantity One one-dimensional analysis software (Bio-Rad). Detailed information regarding the sources and dilutions of the primary antibodies can be found in supplementary Table S3.

### 2.8 Detection of glycolytic activity

**Lactate assay:** Lactate levels were determined via the CheKine™ Micro Lactate Assay Kit (Abbkine, Wuhan, China, KTB1100). To prepare articular cartilage tissues, they were homogenized with lactate assay buffer at a 1 mL to 0.1 g tissue ratio on ice, followed by centrifugation for 5 minutes at 12,000 × g and 4 °C. The resulting supernatant was collected for further analysis. For chondrocytes grown in culture, the cells were rinsed with cold PBS, homogenized in lactate assay buffer (1 mL per 5 million cells) while on ice, and subjected to ultrasonic disruption (20% power or 200 W, with 3 seconds of sonication followed by 7 seconds of rest, for a total of 30 repetitions). The homogenate was centrifuged at 12,000 × g for 5 minutes at 4 °C, and the supernatant was extracted for measurement. The samples were then combined with working reagent and incubated at 37°C for 30 minutes in a dark environment. Absorbance readings were taken at a wavelength of 450 nm.

**Extracellular acidification rate (ECAR) detection:** The ECAR was assessed via an ECAR fluorometric assay kit (Elabscience, Wuhan, China; E-BC-F069). Chondrocytes were plated at a density of 10⁵ cells per well in a black 96-well plate and cultured overnight. Following the experimental design, the culture medium was gently aspirated and replaced with 200 µL of the probe working solution. To measure background fluorescence, blank wells containing only the probe working solution without cells were included. The fluorescence was recorded every three minutes over a span of 100 minutes at excitation/emission wavelengths of 490/535 nm via a fluorescence microplate reader. The calculation of the ECAR was based on the linear segment of the fluorescence-time curve.

**Glucose uptake assay:** Glucose uptake was assessed via the Glucose Uptake Fluorometric Assay Kit (Elabscience, E-BC-F041). Chondrocytes were cultivated at a density of 2,000 cells per well in 96-well plates and treated as needed. After an overnight serum starvation period, the cells were rinsed and incubated with 100 µL of KRPH buffer containing 2% BSA. Next, 10 µL of 10 mM 2-deoxyglucose (2-DG) was delivered to the test wells, whereas the control wells received 10 µL of KRPH buffer instead. After a 30-minute incubation at 37°C, the cells were washed three times with KRPH buffer. Next, 50 µL of Reagent I was added to each well, followed by the addition of 50 µL of Reagent II after a 10-minute period at RT. Standards and samples (30 µL each) were transferred to a fluorescence-compatible plate, combined with 170 µL of the working solution, and incubated at 37 °C for another 30 minutes. The fluorescence was recorded at 530/590 nm (excitation/emission).

**ATP production measurement:** The ATP levels were assessed via an ATP chemiluminescence assay kit (Elabscience, E-BC-F002). We lysed a total of 2 × 10⁶ pretreated chondrocytes in 0.3 mL of Reagent I, followed by heating in a boiling water bath for 10 minutes. The samples were then cooled on ice and centrifuged at 10,000 × g for 10 minutes at a temperature of 4°C. The resulting supernatant was collected for subsequent analysis. To each well of a luminescence plate, a 100 µL aliquot of the working solution was introduced and equilibrated for 5 minutes. Subsequently, 100 µL of either the standard or the supernatant sample was added and mixed immediately. Chemiluminescence readings were obtained via a luminescence detector.

### 2.9 Cell immunofluorescence

Chondrocytes were placed on glass coverslips in 6-well plates. After a period of 24 hours, the cells were treated in accordance with the experimental protocol. Following the treatments, the cells were fixed with 4% paraformaldehyde for 15 minutes at RT and subsequently washed twice with PBS. For blocking, QuickBlock™ Blocking Buffer for Immunol Staining (Beyotime, P0260) was applied for 10 minutes at RT. The cells were then incubated overnight at 4°C with primary antibodies that had been diluted in an antibody dilution buffer, followed by a 1-hour incubation at RT with appropriate fluorophore-conjugated secondary antibodies shielded from light. Nuclei were stained with DAPI (Beyotime, P0131) for 5 minutes. After the final washes were complete, the coverslips were mounted on glass slides using antifade mounting medium. Images were captured via a fluorescence microscope equipped with suitable filter sets.

### 2.10 Histopathological examination

**Routine pathological examination:** Following the anesthetic procedure, the knee joints of the rats were dissected and preserved in 4% paraformaldehyde for one night. The tissues were decalcified with EDTA until they were sufficiently softened. Upon completing decalcification, specific areas of the tissues were trimmed and positioned into embedding cassettes. The samples were subjected to dehydration through a series of graded ethanol concentrations, subsequently cleared with xylene, and embedded in paraffin. Using a microtome, sections of 4 µm thickness were obtained. These sections were dried overnight in an oven set at 60 °C and were kept at RT. For the purpose of histological analysis, the sections were stained with hematoxylin and eosin (H&E) (Servicebio, G1076), Masson's trichrome (Servicebio, G1006), or Safranin O-Fast Green (SO-FG, Servicebio, G1053) in accordance with the instructions provided by the manufacturer.

**Immunohistochemistry (IHC) staining:** Sections that had been deparaffinized and rehydrated were subjected to antigen retrieval with citrate buffer (pH 6.0), which was heated in a water bath. Endogenous peroxidase activity was inhibited by incubating the sections in 3% hydrogen peroxide for 25 minutes at RT in the absence of light. The sections were subsequently blocked with 3% BSA for 30 minutes at RT and then incubated overnight at 4°C with primary antibodies that had been diluted in blocking buffer. Following washing, HRP-conjugated secondary antibodies were added for 50 minutes at RT. A DAB substrate kit was used to develop the signal until the desired staining intensity was achieved. Hematoxylin was used to counterstain the nuclei. The staining was visualized and captured via a light microscope.

### 2.11 Cell counting kit-8 assay

Cell growth was evaluated via the Cell Counting Kit-8 (CCK-8; Dojindo, Kumamoto, Japan, CK04). In brief, chondrocytes were plated in 96-well plates at a density of 5 × 10³ cells per well and treated as specified. Following incubation for predetermined time periods, 10 µL of the CCK-8 reagent was added to each well and then incubated for 2 hours at 37°C. The optical density was recorded at 450 nm via a microplate reader.

### 2.12 Transwell migration assay

Cell migration was assessed via Transwell chambers (Corning, USA). Chondrocytes that had been pretreated were resuspended in medium containing 1% FBS and placed in the upper chamber. The lower chamber received complete medium supplemented with 10% FBS, which served as a chemoattractant. After incubation for 24 h, any nonmigrating cells adhering to the upper membrane surface were carefully removed with a cotton swab. The cells that successfully migrated to the lower surface were fixed with 4% paraformaldehyde and subjected to staining with 0.1% crystal violet (Beyotime, China) for 15 minutes. A light microscope (Olympus IX70) was used to capture images, and the number of migrated cells was quantified across three randomly chosen fields per well.

### 2.13 Scratching assay

Chondrocytes were placed in 6-well plates and grown until they reached 90-100% confluence. Following a 48-hour pretreatment period, the culture medium was changed to serum-free medium. A sterile 200 µL pipette tip was used to generate a consistent wound across the cell layer. The wells were carefully rinsed with PBS to eliminate any detached cells or debris. Wound healing was observed at 0, 24, and 48 hours after scratching, and images were captured via a light microscope (Olympus IX70).

### 2.14 Cell high-density micromass culture and Alcian blue staining

To generate chondrocyte micro-aggregates, 1.5×10⁵ cells in 10 μL of DMEM/F12 medium were plated per well (24-well plate) and allowed to adhere for 1 h at 37°C. Then, 500 μL of DMEM/F12 medium containing 10 ng/mL ITS and 2% FBS was added. Cultures were maintained with medium changes every other day for 14 days, followed by Alcian blue staining (Solarbio, Shanghai, China, G1565) at RT. The pretreated chondrocytes were fixed with methanol for 5 minutes at RT and subsequently rinsed with chilled distilled water. The cells were then stained with Alcian blue solution A for 30 minutes at RT, followed by two washes with chilled distilled water. After that, the cells were counterstained with Alcian blue solution for 30-60 s, gently rinsed with distilled water, and observed under a light microscope (Olympus IX70) while still moist to avoid drying.

### 2.15 Senescence-associated β-galactosidase (SA-β-Gal) detection

Cellular senescence was evaluated via a β-galactosidase staining kit (Beyotime, C0602). In summary, chondrocytes were fixed with SA-β-Gal staining fixative for 15 minutes at RT. Following the removal of the fixative, the cells were rinsed three times with PBS, with each rinse lasting 3 minutes. A working solution for staining, formulated in accordance with the manufacturer's guidelines, was subsequently applied to the cells. To prevent evaporation, the plates were covered with parafilm and incubated overnight at 37 °C in a dry incubator that was free of CO₂. Images were captured via a light microscope (Olympus IX70).

### 2.16 Enzyme-linked immunosorbent assay (ELISA) for SASP detection

Increased production of SASP factors is a defining characteristic of cellular senescence. To assess the expression of SASP factors, the concentrations of IL-6, IL-10, MMP13, and MMP3 were quantified via commercial ELISA kits (Elabscience: E-EL-R0015, E-EL-R0016, E-EL-R0045, and E-EL-R0619) following the guidelines provided by the manufacturer. Chondrocytes were carefully washed with cold PBS and subsequently detached with trypsin. Following centrifugation at 1000 × g for 5 minutes, the cell pellets were resuspended in 200 μL of PBS containing 1% PMSF for every 10⁶ cells, followed by ultrasonic disruption. The lysate was centrifuged at 1500 × g for 10 minutes at 4 °C to eliminate debris. All reagents, which included wash buffer, standard solutions, biotinylated detection antibodies, and HRP-conjugated working solutions, were prepared according to the instructions. Standards and samples were applied to precoated plates, which were subsequently sealed and incubated at 37 °C for 90 minutes. After incubation, the liquid was carefully removed without washing. Next, 100 μL of biotinylated antibody working solution was added to each well, after which the plate was resealed and incubated at 37 °C for an additional hour. The plate was then washed three times with 350 μL of wash buffer per well, allowing for a 1-minute soak. Next, 100 μL of HRP enzyme conjugate working solution was dispensed into each well, and the plate was covered and incubated at 37 °C for 30 minutes. After another washing cycle, 90 μL of TMB substrate solution was added to each well, and the plate was incubated in the dark at 37 °C for 15 minutes. The reaction was halted by adding 50 μL of stop solution per well, and the absorbance was promptly measured at 450 nm via a microplate reader.

### 2.17 CUT&TAG sequencing

CUT&TAG sequencing was performed by Novogene Co., Ltd. (Beijing, China). Articular cartilage samples were snap-frozen in liquid nitrogen and stored at -80 °C. For nuclear extraction, 1 mL of 1× HB buffer was added to the frozen tissue, which was then ground, filtered, and centrifuged at 500 × g for 5 minutes at 4°C. The pellet was subjected to density gradient centrifugation for 10 minutes. Nuclei were resuspended, counted using a LUNA-FL automated cell counter, and used for subsequent steps. The CUT&TAG assay was carried out via the CUT&TAG Assay Kit (Novogene) following the manufacturer's protocol. Briefly, the pA-Tn5 transposase was guided by a specific antibody to bind genomic regions associated with the target protein, simultaneously adding sequencing adapters to both ends of the cleaved DNA. The library was constructed via PCR amplification. After PCR, the libraries were purified via AMPure XP beads, and their quality was assessed via an Agilent Bioanalyzer 2100 system. Sequencing was performed on the Illumina NovaSeq platform to generate 150 bp paired-end reads. The raw sequencing reads were quality-processed with fastp (v0.20.0). Clean reads were aligned to the reference genome via BWA (v0.7.12). Peak calling was conducted with MACS2 (v2.1.0) under default parameters (q value < 0.05). Peaks were annotated and associated with genes via ChIPseeker. Functional enrichment analyses for GO terms and KEGG pathways were performed via clusterProfiler and KOBAS, respectively. Visualization of the peaks was carried out with IGV (v2.14.1).

### 2.18 Dual-luciferase assay

Cells in a healthy growth state were seeded into 24-well plates one day prior to transfection. Transfection was performed using the X-tremegene HP reagent (ROCHE, 06366236001). Per well, 1 μg of plasmid and 2 µL of transfection reagent were combined in 100 µL of opti-MEM medium (GIBCO, 31985-070), mixed, and incubated at RT for 20 min to form complexes. Meanwhile, the culture medium in the plates was replaced with 200 µL of opti-MEM. The complex solution was then added to the cells. After 5-6 h of incubation at 37°C with 5% CO₂, the medium was replaced with fresh complete medium supplemented with 10% serum. At 24 h post-transfection, transfection efficiency was initially assessed by observing fluorescence. Cells were subsequently lysed and analyzed using the Dual-Luciferase® Reporter Assay System (E1910, Promega) according to the manufacturer's instructions.

### 2.19 Chromatin immunoprecipitation (ChIP) assay

ChIP assays were performed using the BeyoChIP™ ChIP Assay Kit (Beyotime, P2078). Briefly, cells were cross-linked by adding formaldehyde directly to the culture medium (final concentration 1%) and incubating at 37 °C for 10 min. The reaction was quenched with Glycine Solution for 5 min at RT. After discarding the supernatant, cells were washed twice with ice-cold PBS containing 1 mM PMSF. Cell pellets were collected by centrifugation, resuspended in SDS Lysis Buffer supplemented with 1 mM PMSF, and incubated on ice for 10 min to ensure complete lysis. The genomic DNA was then fragmented by ultrasonication. Following sonication, samples were centrifuged and the supernatant was collected. ChIP Dilution Buffer containing 1 mM PMSF was added to the supernatant. To pre-clear the lysate, Protein A+G Agarose/Salmon Sperm DNA was added and incubated with gentle rotation at 4 °C for 30 min. The pre-cleared lysate was then incubated with primary antibody or control IgG overnight at 4 °C with gentle agitation. Protein A+G Agarose/Salmon Sperm DNA was added again and incubated for 60 min at 4 °C to capture the antibody-bound complexes. After centrifugation, the beads were collected and washed sequentially. The immunoprecipitated DNA was purified using a DNA purification kit (Beyotime, D0033) and analyzed by qPCR.

### 2.20 Transmission electron microscopy for autophagy

For the ultrastructural examination of autophagy, chondrocytes were harvested via centrifugation and subsequently fixed with ice-cold electron microscopy-grade glutaraldehyde for 4 hours. After the fixation process, the cells were pelleted, and the excess supernatant was carefully discarded. The cell pellet was washed three times with 0.1 M phosphate buffer (PB, pH 7.4). A slightly cooled 1% agarose solution was used to embed the pellet until solidification occurred. The agarose-embedded samples were subsequently fixed in a 1% osmium tetroxide solution in 0.1 M PB for 2 hours at RT in the absence of light. The samples were subsequently dehydrated through a serial gradient of ethanol (50%, 70%, 80%, 90%, and 100%) for 20 minutes at each concentration and then subjected to two exchanges of 100% acetone for 15 minutes each. The infiltration was performed using a 1:1 blend of acetone and EPON 812 resin overnight at 37°C, followed by exposure to pure resin for 5--8 hours. Finally, the samples were embedded in fresh EPON 812 resin and polymerized at 60°C for 48 hours. Ultrathin sections (60-80 nm) were cut with an ultramicrotome and placed onto 150-mesh copper grids. The sections were stained with 2% uranyl acetate diluted in 50% ethanol for 8 minutes in the dark, followed by further staining with 2.6% lead citrate for an additional 8 minutes. Imaging was conducted via transmission electron microscopy, and the resulting images were analyzed for autophagic structures.

### 2.21 Autophagy flux detection

Autophagic flux was monitored via the mRFP-GFP-LC3B fluorescence system. Chondrocytes were seeded into 24-well plates and infected with a recombinant adenovirus encoding mRFP-GFP-LC3B (Hanbio Biotechnology, HBAP2100001; MOI = 40) for 24 hours. After viral transduction, the cells were subjected to the indicated treatments. Fluorescence images were acquired via a fluorescence microscope. Autophagosomes were identified as yellow puncta (positive for both mRFP and GFP signals), whereas autolysosomes appeared as red puncta (mRFP-positive only due to GFP quenching in acidic compartments). The numbers of autophagosomes and autolysosomes were quantified, and their ratios were calculated to evaluate alterations in autophagic flux.

### 2.22 Molecular docking

Molecular docking was performed via the HDOCK server to predict the binding interactions between TRIM29 and the subunits of PI3Ks (PIK3CA, PIK3CB, PIK3R1, and PIK3R2) and EGFR. The fast Fourier transform algorithm was employed for global conformational sampling of possible binding modes. A knowledge-based scoring function (ITScorePP/ITScorePR) was used to evaluate the generated poses. The top 10 docking models were visually inspected, and the top 100 results were made available for download. The model with the most negative docking score was selected for further analysis. Protein-protein interactions were visualized and analyzed via PyMOL. Docking scores were computed iteratively; more negative values indicate stronger binding affinity. A docking score below -200 and a confidence score above 0.7 were considered indicative of high-probability binding.

### 2.23 Coimmunoprecipitation (Co-IP) analysis

Co-IP experiments were performed via an immunoprecipitation kit with protein A+G magnetic beads (Beyotime, P2179S). For each sample containing 1 million cells, 200 μL of ice-cold lysis buffer containing protease inhibitors was used for cell lysis, and 600 μL of the same buffer was prepared for subsequent washing steps. The magnetic beads were gently resuspended, and 20 μL of bead suspension was used per 500 μL of sample. An appropriate volume of beads was transferred to a clean tube, washed with TBS, and separated via a magnetic stand. Total protein was extracted via inhibitor-supplemented lysis buffer and clarified via centrifugation on ice. Antibody working solutions were prepared in TBS according to the manufacturer's recommended dilution. Protein A+G magnetic beads were incubated with 500 μL of antibody solution or normal IgG control for 30 minutes at RT with rotation. The beads were then washed with 500 μL of TBS and resuspended. The antibody-bound beads were added to the protein lysates and incubated overnight at 4°C with rotation. After incubation, the beads were washed once with 0.5 mL of inhibitor-containing lysis buffer. To elute the bound proteins, 100 μL of SDS‒PAGE sample loading buffer was added per 20 μL of original bead mixture, followed by heating at 95°C for 5 minutes. The eluate was separated magnetically, and the supernatant was collected for western blot analysis.

### 2.24 Statistical analysis

All experiments were performed in at least three independent replicates. The data are expressed as the means ± standard deviations. Statistical comparisons between groups were carried out via t tests or one- or two-way analysis of variance (ANOVA) followed by Tukey's post hoc test for multiple comparisons, as appropriate. All the analyses were conducted via GraphPad Prism version 8.0. A p value < 0.05 was considered statistically significant.

## 3. Results

### 3.1 Chondrocyte Glycolysis is Elevated in OA

**Figure 1:**
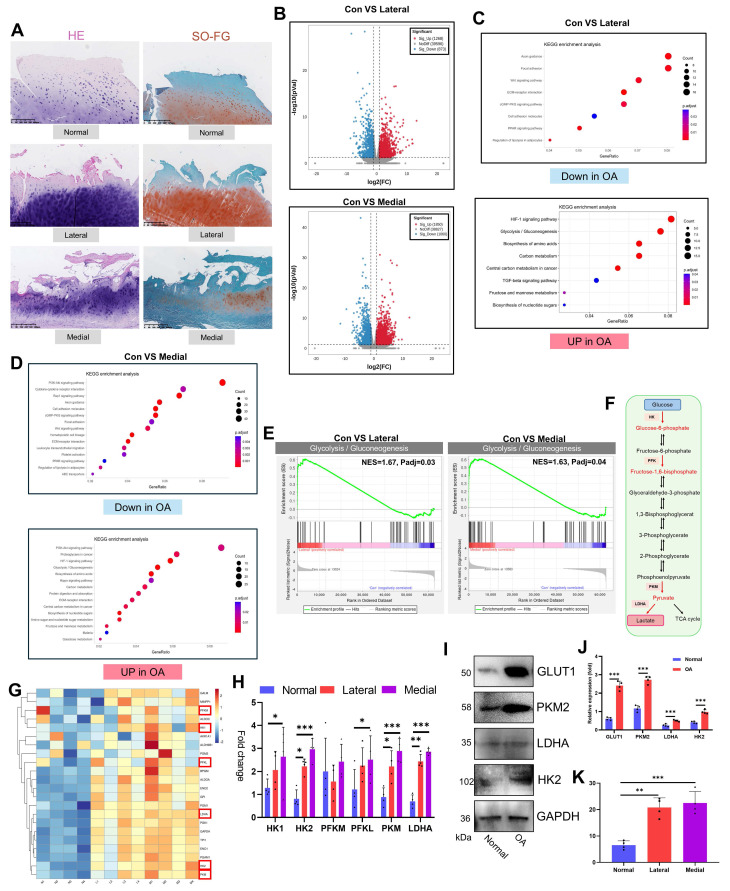
Glycolysis is increased in the articular cartilage of OA patients. A) Histopathological examination (H&E and SO-FG staining) of articular cartilage. B) Volcano plot of transcriptomic sequencing data from articular cartilage. C, D) KEGG pathway enrichment analysis of upregulated and downregulated genes in OA articular cartilage (including the medial and lateral compartments). E) GSEA for glycolysis/gluconeogenesis. F) Schematic diagram of the glycolytic pathway. G) Heatmap displaying the expression of genes involved in the glycolysis/gluconeogenesis pathway across articular cartilage samples. H) Expression levels of key glycolytic enzymes in individual articular cartilage samples (n=4). I, J) Western blot analysis and semi-quantification of key glycolytic enzymes in articular cartilage from healthy donors and OA patients (n=4). K) Lactate content in the articular cartilage (n=4). *P < 0.05, **P < 0.01, ***P < 0.001.

To investigate OA pathology, we obtained damaged articular cartilage from patients undergoing TKA for OA and normal cartilage from patients undergoing ACLR. Macroscopic evaluation revealed significant surface damage and osteophyte hyperplasia in OA cartilage (**Fig. S1A**). Histological analysis via H&E and SO-FG staining demonstrated marked differences. Healthy cartilage exhibited a smooth, intact surface without fissures or tissue hyperplasia. In contrast, OA cartilage (both the medial and lateral compartments) displayed severe degeneration, including surface disruption and significant cartilage layer thinning (**Fig. 1A**). Transcriptome sequencing of articular chondrocytes revealed pronounced gene-level alterations in OA. Differential expression analysis (|log₂FC| > 1, p<0.05) revealed significant changes: 1,268 upregulated and 873 downregulated genes in OA lateral cartilage versus healthy tissue and 1,850 upregulated and 1,060 downregulated genes in OA medial cartilage versus healthy tissue (**Fig. 1B, S1B**). KEGG enrichment analysis of differentially expressed genes (DEGs) highlighted key pathways. The upregulated DEGs were enriched primarily in glycolysis/gluconeogenesis, the HIF-1 signaling pathway, carbon metabolism, and central carbon metabolism in cancer. The downregulated DEGs were enriched in focal adhesion, the Wnt signaling pathway, the ECM-receptor interaction, the cGMP-PKG signaling pathway, and the PPAR signaling pathway (**Fig. 1C, D**). GO analysis further corroborated the enrichment of upregulated DEGs in glycolysis-related terms (**Fig. S1C, D**). Consistent with these findings, GSEA confirmed significant upregulation of glycolysis/gluconeogenesis in both medial and lateral OA cartilage (**Fig. 1E**). Direct examination of glycolysis pathway genes revealed elevated expression of key enzymes (HK1, HK2, PFKL, PKM, and LDHA) in OA cartilage (PFKM was unchanged) (**Fig. 1G, H**). Western blot analysis confirmed the increased protein levels of the glycolysis markers GLUT1, PKM2, LDHA, and HK2 in the OA samples (**Fig. 1I, J**). Furthermore, the levels of lactate, the metabolic end-product of glycolysis, were significantly elevated in OA cartilage (**Fig. 1K**). Overall, our comprehensive analysis of clinical samples established enhanced glycolytic activity as a central feature of OA pathogenesis.

**Figure 2:**
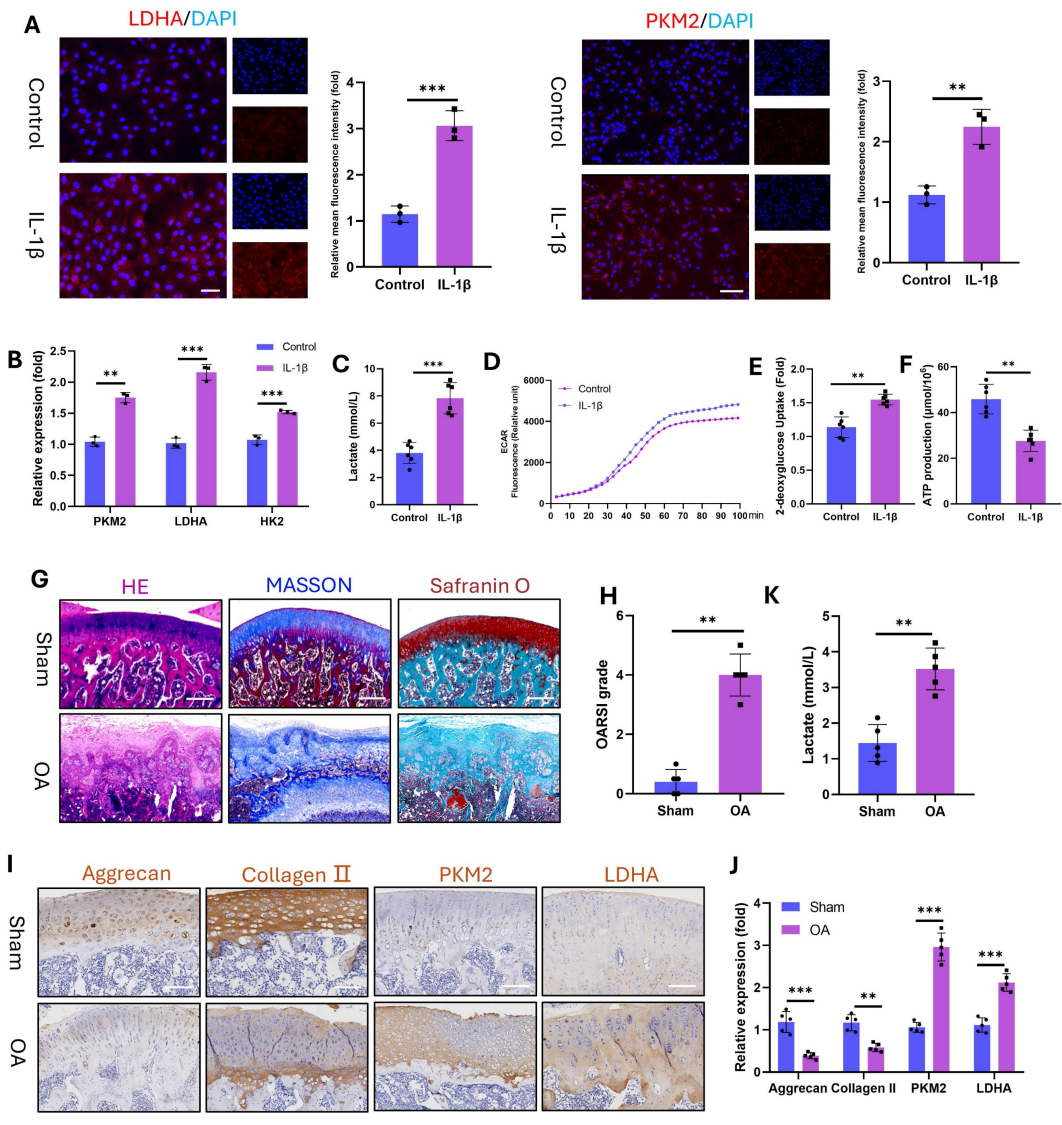
Glycolytic activity is enhanced in IL-1β-stimulated (10 ng/mL) chondrocytes and in a rat model of OA. A) Immunofluorescence staining of LDHA and PKM2 in chondrocytes stimulated with IL-1β for 24 h (n=3). Scale bar, 100 μm. B) mRNA expression levels of key glycolytic enzymes (PKM2, LDHA, and HK2) measured by qPCR for 24 h (n=3). C) Lactate production in chondrocytes stimulated with IL-1β for 24 h (n=3). D) Dynamic changes in the ECAR over 100 min in IL-1β-stimulated chondrocytes. E) Glucose uptake capacity of IL-1β-stimulated chondrocytes after 24 h (n=3). F) ATP synthesis ability of IL-1β-stimulated chondrocytes after 24 h (n=3). G) Histopathological evaluation of articular cartilage morphology in the SD rat OA model at 12 weeks (n=5). Scale bar, 100 μm. H) OARSI score of the articular cartilage in the SD rat OA model (n=5). I) Lactate content in the articular cartilage of SD model rats (n=5). Scale bar, 100 μm. J, K) IHC analysis of Aggrecan, Collagen II, PKM2, and LDHA expression in articular cartilage (n=5). **P < 0.01, ***P < 0.001.

Furthermore, OA is a chronic inflammatory disorder^27^. To model this pathology in vitro, we stimulated chondrocytes (marked with Collagen II, **Fig. S2**) with IL-1β and assessed glycolytic activity. IL-1β significantly upregulated the protein expression of the glycolytic enzymes LDHA and PKM2 (**Fig. 2A**) and the mRNA levels of LDHA, PKM2, and HK2 (**Fig. 2B**). Concomitantly, the level of intracellular lactate, a terminal glycolytic product, increased in the IL-1β-treated chondrocytes (**Fig. 2C**). This elevated lactate secretion augmented the ECAR, as demonstrated by real-time metabolic profiling (**Fig. 2D**). Enhanced glycolytic flux reduces ATP production while increasing glucose demand. Consistent with these findings, IL-1β-stimulated chondrocytes presented decreased ATP (**Fig. 2E**) and elevated 2-deoxyglucose uptake (**Fig. 2F**). To corroborate these findings in vivo, we induced OA in SD rats via ACLT. Histopathology revealed severe articular cartilage degeneration in the ACLT joints, characterized by surface fibrillation, cartilage thinning, and subchondral bone hyperplasia (**Fig. 2G**). OA progression was quantified by significant increases in OARSI scores (**Fig. 2H**). IHC revealed reduced expression of chondroprotective proteins (Aggrecan and Collagen II) but increased expression of glycolytic enzymes (PKM2 and LDHA) in OA cartilage (**Fig. 2I, J**). Joint lactate levels were also significantly greater in OA rats than in control rats (**Fig. 2K**). These data establish that OA pathogenesis involves glycolytic hyperactivation in chondrocytes, which is evident both in vitro under inflammatory challenge and in vivo in ACLT-induced OA. This finding aligns with clinical observations (the first section), confirming that glycolysis is a key pathological factor of OA progression.

### 3.2 Excessive glycolytic lactate accelerates chondrocyte senescence

**Figure 3:**
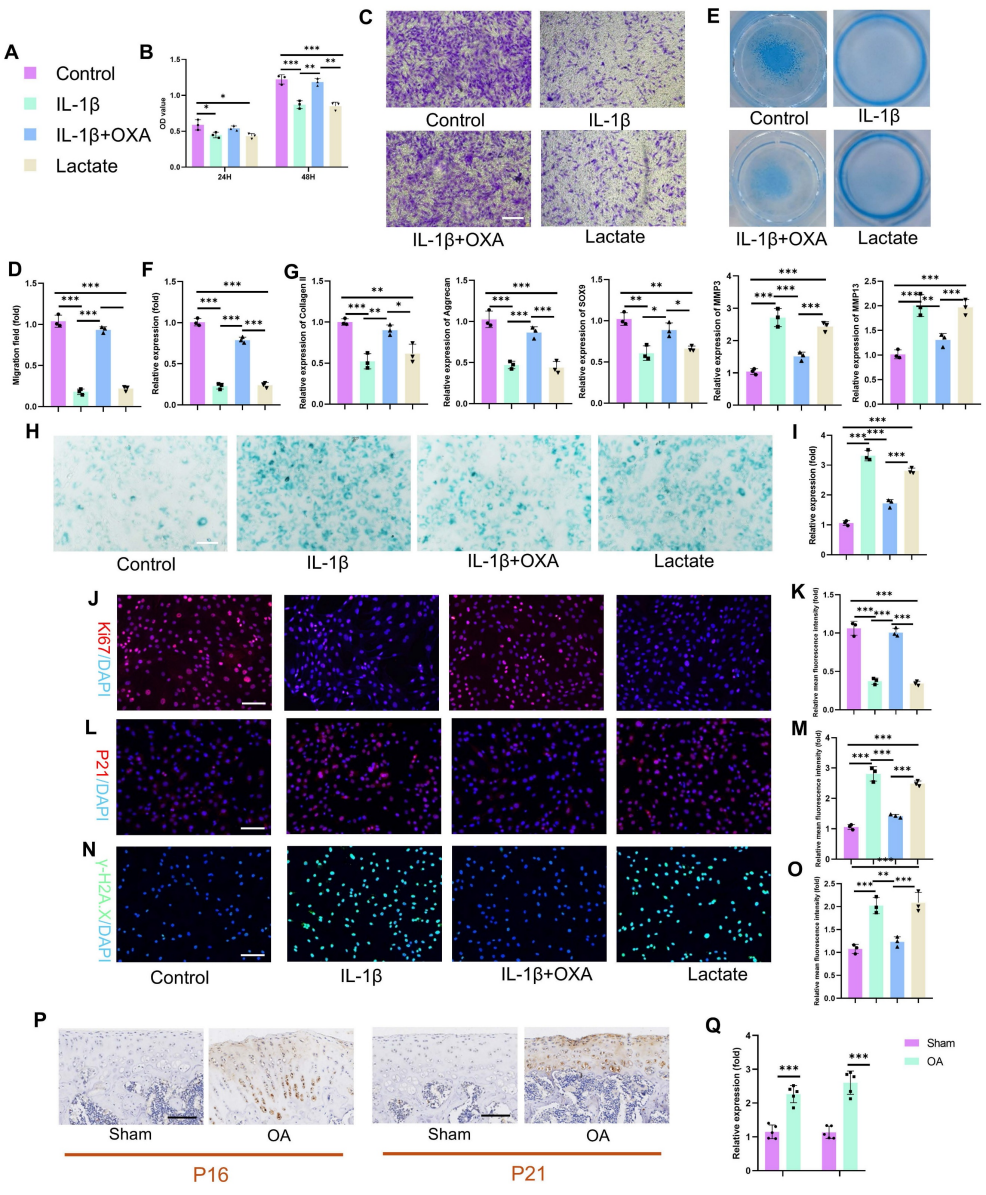
Excess lactate derived from glycolysis induces chondrocyte senescence. A) Illustration of color representations used in the subsequent panels. B) Proliferative capacity of chondrocytes treated with or without lactate (20 mM) (n=3). C, D) Transwell assay evaluating the migratory ability of chondrocytes treated with IL-1β (10 ng/mL), lactate (20 mM) or OXA (10 μM) for 24 h. The same treatment protocol was used for the following experiments (n=3). Scale bar, 100 μm. E, F) Chondrogenic capacity of chondrocytes, as assessed by cell high-density micromass culture and Alcian blue staining (n=3). Scale bar, 100 μm. G) mRNA expression levels of Collagen II, Aggrecan, SOX9, MMP3, and MMP13 measured by qPCR (n=3). H, I) SA-β-Gal staining of chondrocytes to assess senescence activity (n=3). Scale bar, 100 μm. J, K) Immunofluorescence staining of Ki67 expression in chondrocytes (n=3). Scale bar, 100 μm. L, M) Immunofluorescence detection of P21 expression in chondrocytes (n=3). Scale bar, 100 μm. N, O) Immunofluorescence analysis of γ-H2AX expression in chondrocytes (n=3). Scale bar, 100 μm. P, Q) IHC analysis of senescence markers (P16 and P21) in articular cartilage from the SD rat OA model (n=5). Scale bar, 100 μm. *P < 0.05, **P < 0.01, ***P < 0.001.

The specific mechanisms by which lactate influences chondrocyte pathophysiology remain incompletely defined. We therefore investigated the functional impact of IL-1β-induced glycolysis and lactate production on chondrocyte biology by employing oxamate (OXA; an LDHA inhibitor) to disrupt lactate generation. IL-1β and exogenous lactate significantly suppressed chondrocyte proliferation (**Fig. 3B**). This effect was reversed by OXA cotreatment, restoring proliferation capacity by 48 hours. Consistent with impaired viability and motility, lactate or IL-1β treatment reduced chondrocyte migration in transwell and scratch assays (**Fig. 3C-D, S3A-B**). OXA intervention mitigated this suppression, rescuing migration capacity. IL-1β and lactate profoundly impair chondrogenic function. Alcian blue staining revealed decreased proteoglycan synthesis and secretion (**Fig. 3E-F**), which was abrogated by OXA. PCR analysis confirmed the concordant downregulation of anabolic genes (Aggrecan, Collagen II, SOX9) and the upregulation of catabolic genes (MMP3, MMP13) following IL-1β or lactate exposure (**Fig. 3G**). SA-β-Gal activity markedly increased in chondrocytes following IL-1β or lactate treatment (**Fig. 3H-I**). This senescence phenotype was attenuated by OXA. Immunofluorescence analysis corroborated these findings: Ki67 (a proliferation marker) decreased, whereas P16 and P21 (senescence markers) increased in response to IL-1β or lactate. OXA treatment restored Ki67 expression and suppressed P16 and P21 induction (**Fig. 3J-O**). In addition, we quantified SASP components (IL-6, IL-10, MMP3, and MMP13) via ELISA. IL-1β or lactate exposure significantly increased SASP secretion. This response was abrogated by OXA cotreatment, confirming lactate-mediated SASP induction in chondrocytes (**Fig. S4**). Critically, enhanced P16 and P21 expressions were recapitulated in vivo within the knee joint cartilage of an SD rat OA model (**Fig. 3P-Q**), confirming the pathophysiological importance of this pathway. These data establish that IL-1β exerts detrimental effects on chondrocyte proliferation, migration, matrix synthesis, and senescence through the enhancement of glycolytic flux and lactate production. The functional equivalence observed between direct lactate exposure and IL-1β treatment mechanistically implicates lactate as the pivotal effector molecule downstream of inflammatory glycolytic reprogramming in chondrocytes.

To establish the functional relationship between glycolysis and senescence, we quantified glycolytic flux and senescence markers in passage 2 (P2) and passage 15 (P15) chondrocytes. P15 chondrocytes exhibited significantly elevated glycolytic activity relative to P2 controls, as evidenced by increased lactate production, ECAR, and 2-deoxyglucose uptake. Conversely, the cellular ATP levels decreased markedly, which was consistent with a metabolic shift toward glycolysis during senescence (**Fig. S5A**). We next inhibited glycolysis in P15 chondrocytes via OXA to prove causality. Postintervention, senescence assays revealed substantial attenuation of senescent phenotypes: SA-β-Gal activity and P21 expression were reduced compared with those in untreated P15 cells (**Fig. S5B-D**). These data demonstrate that glycolytic hyperactivity is a hallmark of chondrocyte senescence and that its suppression directly mitigates senescence progression. Critically, this mechanistic link substantiates our prior finding that lactate overproduction accelerates chondrocyte aging.

### 3.3 H4K12 lactylation (H4K12la) increased tripartite motif-containing protein 29 (TRIM29) transcription to promote chondrocyte senescence

**Figure 4:**
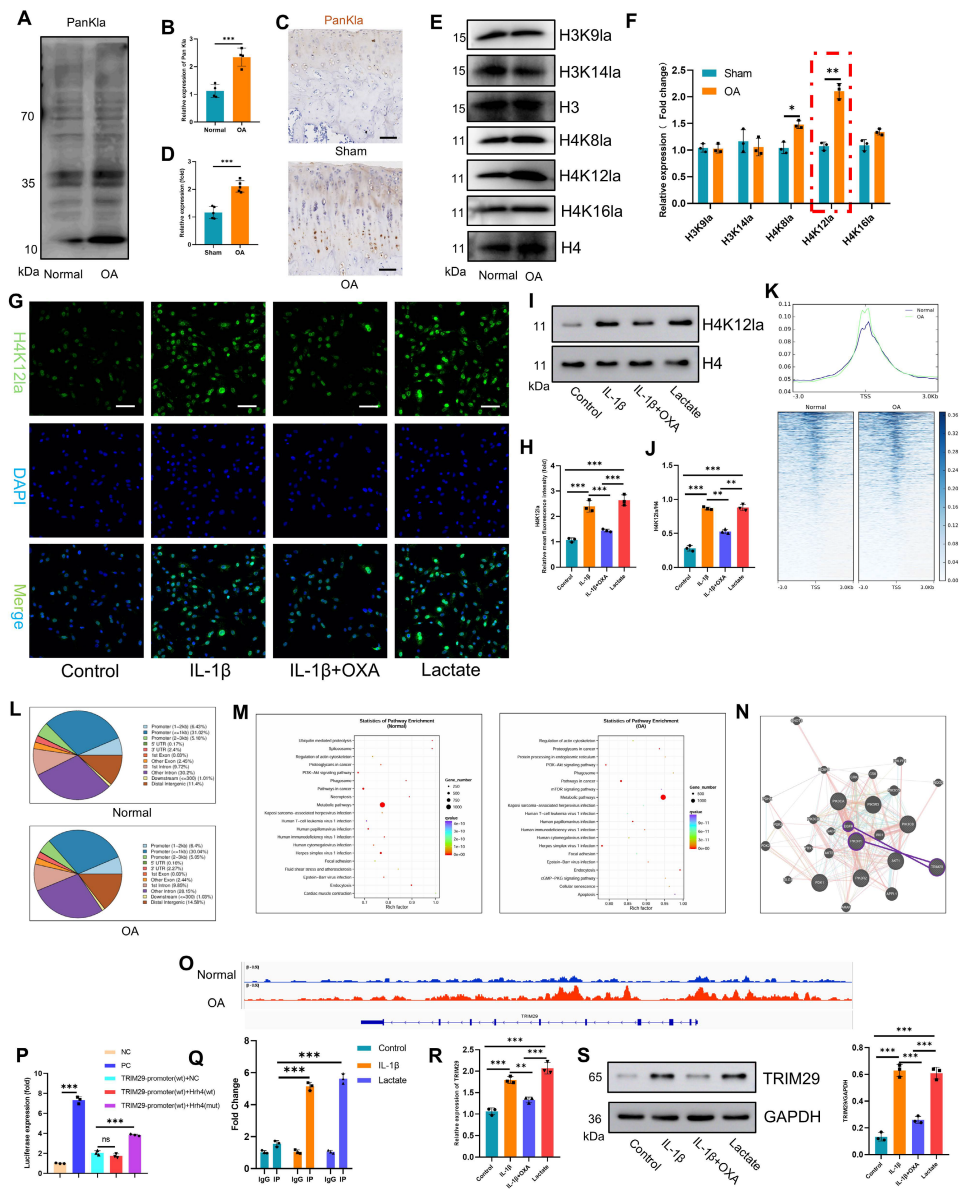
Lactate induces elevated H4K12la levels to enhance TRIM29 transcription. A-D) Western blotting and IHC analysis of PanKla expression in articular chondrocytes (n=4). Scale bar, 50 μm. E, F) Western blotting detection of changes in PanKla expression at various lysine lactylation sites in articular cartilage (n=3). G-J) Immunofluorescence analysis and western blotting analysis of H4K12la levels in chondrocytes (n=3). Scale bar, 100 μm. K) CUT&TAG sequencing analysis showing H4K12la-binding peak density in articular cartilage from healthy donors and OA patients. L) Genomic distribution of H4K12la in articular cartilage from the healthy and OA groups. M) KEGG pathway enrichment analysis of genes associated with H4K12la peaks from CUT&TAG sequencing in the healthy and OA groups. N) GeneMANIA analysis predicting functional interactions between TRIM29 and the PI3K pathway. O) IGV tracks showing enriched CUT&TAG signals for H4K12la at the promoter region of the TRIM29 gene. P) The luciferase assay demonstrated that mutation at the H4K12 site potently activates the TRIM29 promoter. Q) The ChIP assay demonstrated enhanced interaction between H4K12la and the predicted binding site on the TRIM29 promoter after lactate intervention. R, S) qPCR and Western blot analysis of TRIM29 expression at the mRNA and protein levels (n=3). *P < 0.05, **P < 0.01, ***P < 0.001.

Since its discovery in 2019, lactylation has emerged as a key regulator of disease pathogenesis^22^, ^28^, ^29^ and is driven by microenvironmental lactate accumulation. While its role in OA cartilage damage remains unexplored, we detected significantly elevated panlysine lactylation (PanKla) levels in human OA articular cartilage compared with those in normal cartilage (**Fig. 4A, B**), indicating enhanced global lactylation activity. This finding was corroborated in an OA animal model, where immunohistochemistry revealed markedly increased PanKla staining in OA cartilage compared with that in the sham cartilage (**Fig. 4C, D**). The screening of common histone lactylation sites (H3K9la, H3K14la, H4K8la, and H4K16la) revealed H4K12la as the most significantly upregulated mark in human OA cartilage (**Fig. 4E, F**). In vitro, IL-1β and lactate treatment robustly increased PanKla and H4K12la levels in chondrocytes (**Fig. 4G-J, S6**), which were effectively suppressed by the glycolytic inhibitor OXA. These data establish a three-dimensional link between OA pathology, lactate accumulation, and site-specific lactylation (H4K12la).

To define the genome-wide landscape of lactylation-mediated regulation of OA progression, we performed H4K12la-targeted CUT&TAG sequencing on freshly isolated articular cartilage from healthy controls and OA patients. CUT&TAG sequencing demonstrated significant enrichment of H4K12la peaks in OA patient cartilage versus normal control cartilage (**Fig. 4K**). H4K12la was predominantly localized to promoter regions (42.63% and 41.49% in the normal and OA groups, respectively; **Fig. 4L**), indicating direct transcriptional regulation. It suggests that H4K12la in OA may exert its effect by enhancing binding to the promoter region rather than redistributing. GO analysis revealed enrichment of H4K12la-bound "peak genes" in the cellular metabolism and binding pathways (**Fig. S7A**). Crucially, KEGG analysis highlighted differential pathway engagement: the normal group was enriched in the PI3K‒AKT signaling and metabolic pathways, and the OA group was enriched in the PI3K‒AKT signaling, metabolic, mTOR signaling, and cellular senescence pathways (**Fig. 4M**). This directly links lactate or H4K12la to chondrocyte senescence via PI3K-AKT activation.

To identify PI3K‒AKT pathway regulators among H4K12la-bound peak genes, we integrated multiomics signatures from OA cartilage. The integration of OA-upregulated transcriptome genes with H4K12la peak genes identified five candidates (DNER, TRIM29, DAW1, KIF5A, and VSIG10L2; **Fig. S7B**). Interaction network analysis (GENE MANIA) pinpointed TRIM29 as the sole candidate that directly targeted the core PI3K-AKT components EGFR and PIK3R1 (**Figs. 4N, S8**). Integrative Genomics Viewer (IGV) visualization confirmed H4K12la binding within the TRIM29 locus (**Fig. 4O**). We next sought direct evidence for H4K12la binding at the TRIM29 promoter. Luciferase assays revealed that mutating the candidate site abrogated promoter activity (**Fig. 4P**). Corroborating this functional evidence, targeted ChIP-qPCR demonstrated a significant enrichment of H4K12la at the TRIM29 promoter following IL-1β and lactate treatment (**Fig. 4Q**). Similarly, IL-1β and lactate treatment upregulated TRIM29 (**Fig. 4R-S**), whereas OXA suppressed its expression, mirroring lactylation dynamics. Collectively, our data establishes a pathogenic cascade in OA: lactate accumulation drives H4K12 hyper-lactylation, which directly upregulates TRIM29 transcription. TRIM29 activates PI3K-AKT signaling, driving chondrocyte senescence and tissue damage.

**Figure 5:**
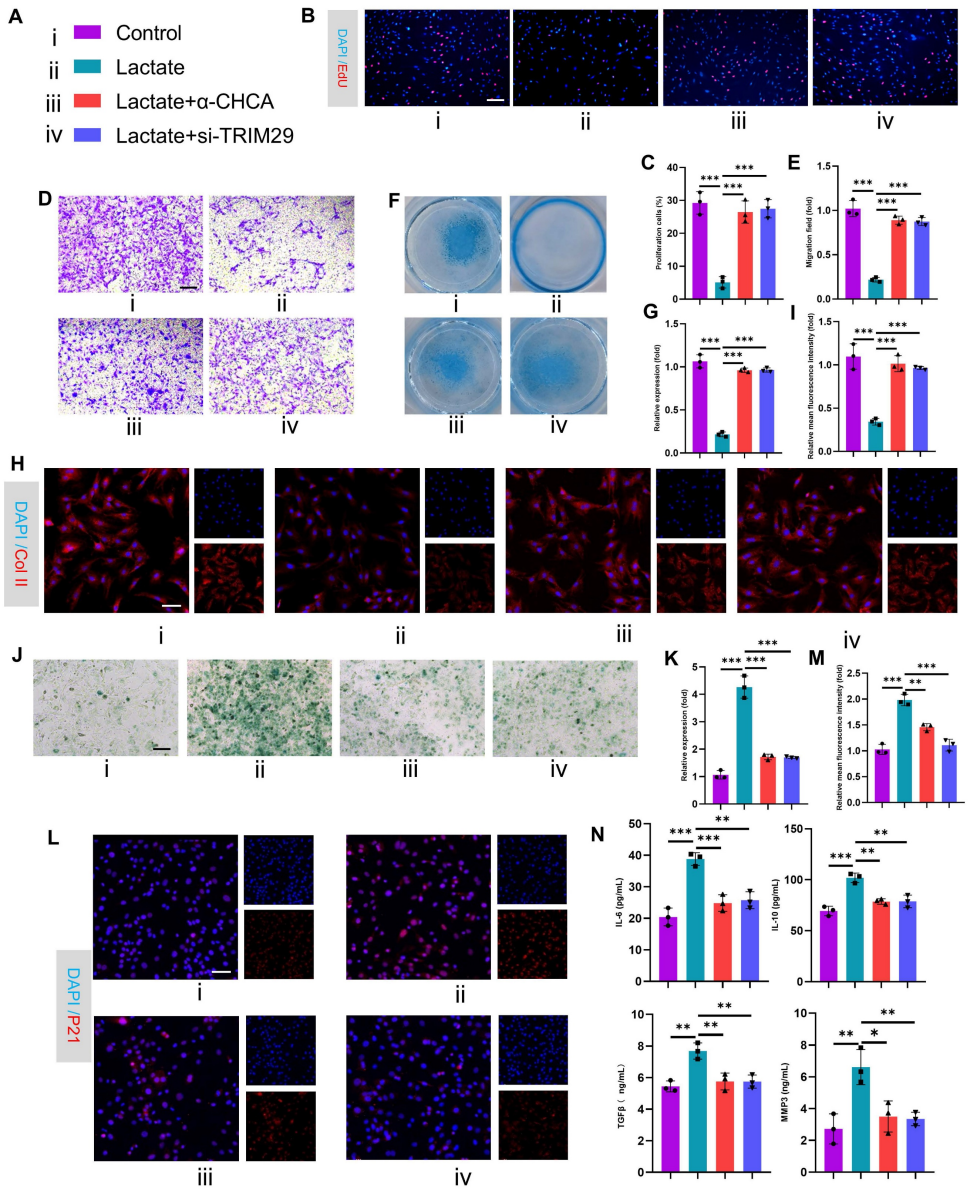
Inhibition of TRIM29 expression alleviates lactate-induced chondrocyte senescence. A) Illustration of color representations used in the subsequent panels. B, C) An EdU assay was used to detect chondrocyte proliferation (n=3). Scale bar, 100 μm. D, E) Transwell assay evaluating chondrocyte migration (n=3). Scale bar, 100 μm. F, G) Chondrogenic capacity was assessed by cell high-density micromass culture and Alcian blue staining (n=3). Scale bar, 100 μm. H, I) Immunofluorescence analysis of Collagen II expression in chondrocytes (n=3). Scale bar, 100 μm. J, K) SA-β-Gal staining indicating senescence levels posttreatment (n=3). Scale bar, 100 μm. L, M) Immunofluorescence detection of P21 expression in chondrocytes after treatment (n=3). Scale bar, 100 μm. N) ELISA analysis of SASP components secreted by chondrocytes following treatment (n=3). *P < 0.05, **P < 0.01, ***P < 0.001.

To validate the pathological role of this mechanism in OA, we performed in vitro functional assays in chondrocytes. First, siRNAs targeting TRIM29 were transfected into chondrocytes to suppress TRIM29 expression, and the knockdown efficiency was quantified by qPCR and Western blotting (**Fig. S9A-B**). Subsequently, EdU assays revealed that lactate treatment suppressed chondrocyte proliferation, whereas cotreatment with α-CHCA or si-TRIM29 reversed this effect (**Fig. 5B-C**). Consistently, transwell assays demonstrated that blocking exogenous lactate uptake or si-TRIM29 intervention partially restored the impaired chondrocyte migration induced by lactate (**Fig. 5D-E**). We next assessed changes in chondrocyte anabolic activity. Alcian blue staining and immunofluorescence revealed that lactate reduced proteoglycan synthesis and collagen II expression, whereas α-CHCA or si-TRIM29 reversed these effects (**Fig. 5F-I**). qPCR further revealed that lactate downregulated anabolic genes (Collagen II, Aggrecan and SOX9) and upregulated catabolic genes (MMP3, MMP13), and these effects were attenuated by α-CHCA or si-TRIM29 (**Fig. S9C**). These results suggest that lactate impairs chondrocyte function via TRIM29 upregulation. To determine whether lactate regulates chondrocyte senescence through TRIM29, SA-β-Gal staining revealed that α-CHCA or si-TRIM29 mitigated lactate-induced senescence (**Fig. 5J-K**). This finding was supported by concordant changes in the senescence marker p21 (**Fig. 5L-M**). Finally, ELISA analysis of SASP components (IL-6, IL-10, MMP3, and MMP13) confirmed that α-CHCA or si-TRIM29 suppressed lactate-induced SASP factor expression (**Fig. 5N**), demonstrating that TRIM29 knockdown attenuated lactate-driven chondrocyte senescence. In summary, these results demonstrated that the inhibition of TRIM29 could mitigate chondrocyte senescence.

### 3.4 TRIM29 dually activates PI3K-AKT signaling to accelerate senescence by suppressing autophagy

**Figure 6:**
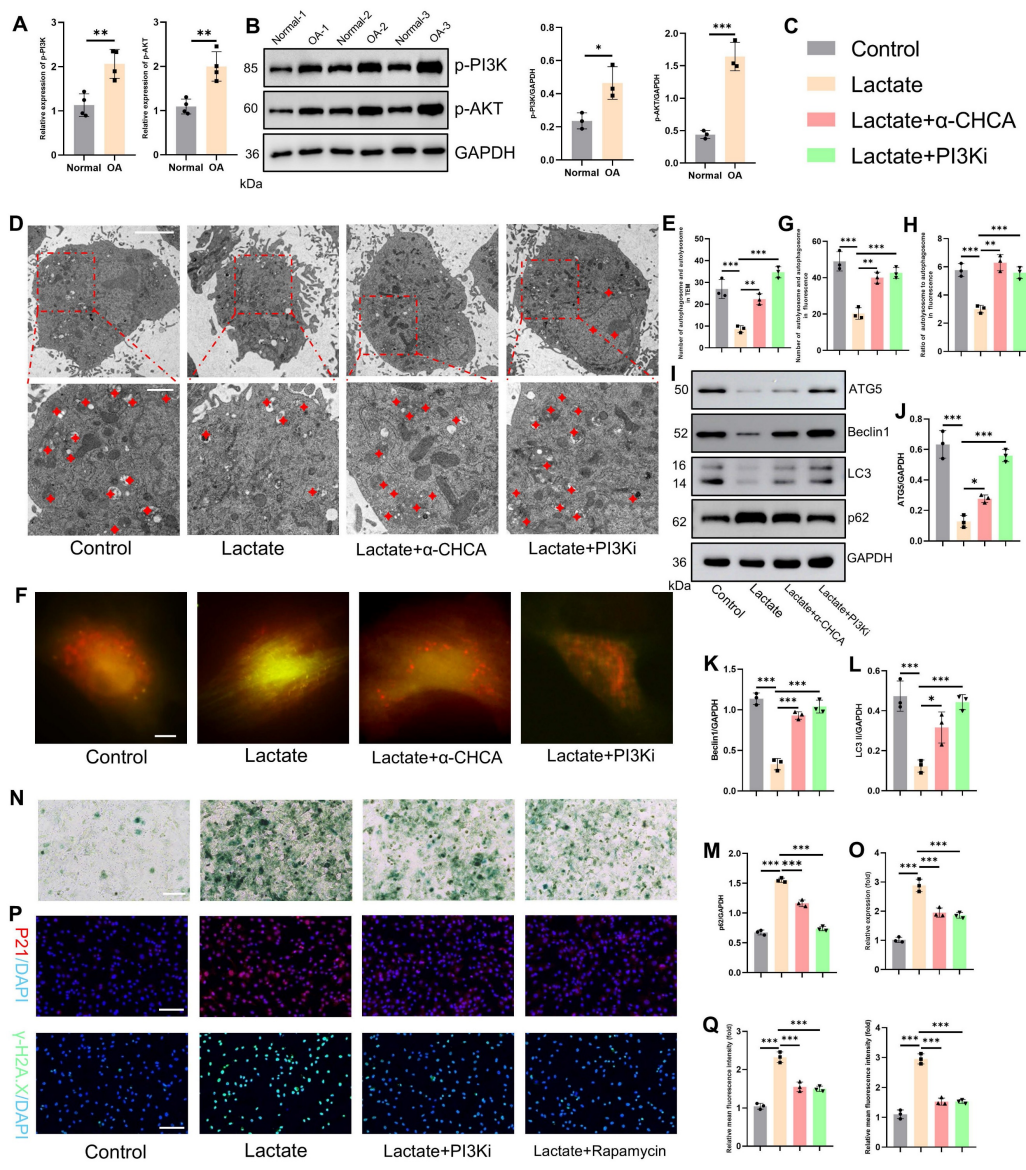
Lactate activates the PI3K-AKT signaling axis to suppress autophagy and promote senescence. A, B) Gene and protein expression levels of p-PI3K and p-AKT in OA articular cartilage (n=3). C) Illustration of color representations used in the subsequent panels. D, E) Number of autophagosomes and autolysosomes (red crosses) in chondrocytes treated with lactate (20 mM), α CHCA (20 μM) or PI3Ki (LY294002, 10 μM) for 24 h, as detected via transmission electron microscopy. The same treatment protocol was used for the following experiments (n=3). Scale bar, 10 μm or 1 μm. F-H) Chondrocytes transfected with mCherry-GFP-LC3 plasmids: yellow puncta indicate autophagosomes, and red puncta represent autolysosomes. (n=3) Scale bar, 10 μm. I-M) Western blot analysis of autophagy markers (ATG5, Beclin1, LC3 and p62). N, O) SA-β-Gal staining of chondrocytes (n=3). P, Q) Immunofluorescence analysis of senescence markers (P21 and γ-H2AX) in chondrocytes (n=3). Scale bar, 100 μm. *P < 0.05, **P < 0.01, ***P < 0.001.

These results establish TRIM29 as a key mediator of lactate-induced chondrocyte senescence, but the precise molecular mechanisms and downstream regulatory pathways involved remain incompletely elucidated. Given the regulatory role of the PI3K-AKT signaling pathway in regulating cell proliferation and differentiation, we first investigated its involvement in OA. Analysis of OA joint cartilage revealed elevated expression of both p-PI3K and p-AKT at the gene and protein levels (**Fig. 6A-B**), indicating activation of this pathway in OA chondrocytes. Furthermore, PI3K-AKT signaling is known to suppress autophagy, a process critical for maintaining cellular homeostasis by clearing damaged components. A decrease in autophagic activity in OA chondrocytes is thought to exacerbate pathology by impairing organelle and protein turnover^30^. Consistent with these findings, transmission electron microscopy revealed that lactate stimulation significantly impaired chondrocyte autophagy, as evidenced by a reduction in the number of autophagosomes and autolysosomes (**Fig. 6D-E**). This impairment was markedly reversed by the inhibition of lactate transport via α-CHCA (an MCT inhibitor) or direct PI3K inhibition with LY294002. To quantify autophagy flux, we employed mCherry-GFP-LC3 reporter assays (yellow puncta: autophagosomes; red puncta: autolysosomes). Lactate treatment dramatically inhibited autophagy flux, which was significantly restored by cotreatment with α-CHCA or LY294002 (**Fig. 6F-H**). Similarly, the expression levels of key autophagy markers (ATG5, Beclin1, LC3-II and p62) followed the same pattern, confirming the suppression of autophagy by lactate and reversal by PI3K pathway inhibition (**Fig. 6I-M**). We subsequently assessed the link between suppressed autophagy and chondrocyte senescence. Concomitant treatment with lactate and either LY294002 or rapamycin (an autophagy enhancer) attenuated the expression of senescence markers, including SA-β-Gal synthesis, and decreased the expression of p21 and γ-H2AX (**Fig. 6O-Q**). To conduct a rescue experiment, autophagy was further inhibited with 3-MA under PI3K pathway blockade. This intervention significantly enhanced chondrocyte senescence, as evidenced by increased SA-β-Gal activity and elevated expression of P21 and γ-H2AX, confirming that autophagy is an essential downstream effector of the lactate-TRIM29-PI3K axis in regulating senescence (**Fig. S10**). These findings demonstrate that inhibiting PI3K signaling or enhancing autophagy counteracts lactate-induced chondrocyte senescence. Collectively, these results indicate that lactate promotes chondrocyte senescence by activating the PI3K-AKT pathway, thereby suppressing autophagy. However, the relationship between TRIM29 and PI3K-AKT signaling remains unclear.

**Figure 7:**
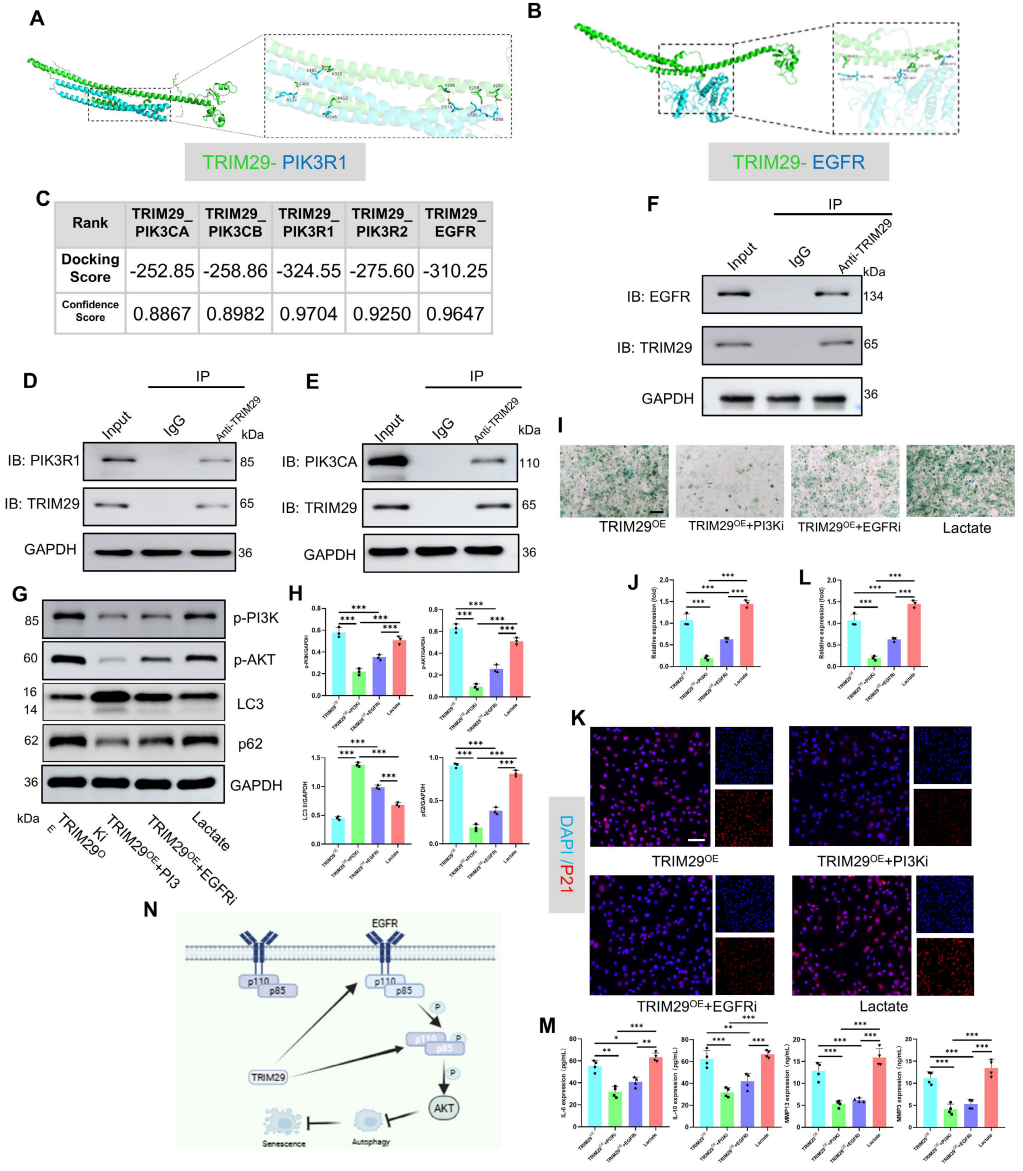
TRIM29 dually activates the PI3K-AKT signaling pathway to promote chondrocyte senescence. A, B) Schematic representation of molecular docking simulations between TRIM29 and PIK3R1 or EGFR. C) Docking scores and confidence scores for the molecular interactions between TRIM29 and PIK3CA, PIK3CB, PIK3R1, PIK3R2, or EGFR. D-F) Co-IP assays detecting the interaction between TRIM29 and PIK3CA, PIK3R1 or EGFR. G, H) Western blotting was used to detect changes in the expression levels of the PI3K-AKT pathway and autophagy activity (n=3). I, J) SA-β-Gal staining showing the effect and regulatory mechanism of TRIM29 overexpression on chondrocyte senescence (n=3). K, L) Immunofluorescence analysis of the senescence marker P21 confirmed the effect and mechanism of TRIM29 overexpression on chondrocyte senescence (n=3). M) ELISA analysis of SASP components in chondrocytes to evaluate the effect and regulatory mechanism of TRIM29 overexpression on cellular senescence (n=3). N) Diagram of TRIM29-mediated dual activation of the PI3K-AKT signaling pathway, which was created via Biorender with permission for publication (n=3). *P < 0.05, **P < 0.01, ***P < 0.001.

Next, as shown in Fig. 5M, TRIM29 was significantly co-expressed with PIK3R1 and EGFR. To elucidate their regulatory relationships, molecular docking simulations were performed to assess the binding sites and free energies between TRIM29 and the PI3K subunits (catalytic: PIK3CAPIK3CB; regulatory: PIK3R1, PIK3R2) or EGFR (**Fig. 7A-C, S11**). Strong binding affinity was defined by a docking score < -200 and a confidence score > 0.7. The results demonstrated high binding affinities for all the tested interactions (**Fig. 7A-C**). Co-IP assays further confirmed direct binding between TRIM29 and PI3K subunits (PIK3CA and PIK3R1) or EGFR (**Fig. 7D-F**), indicating that TRIM29 promotes PI3K‒AKT pathway activation through these interactions. Given that EGFR (a receptor tyrosine kinase, RTK) activates PI3K signaling upon ligand binding^31^, we detected the expression of the PI3K-AKT pathway after TRIM29 overexpression (**Figs. 7G-H, S12**). These results proved that the inhibition of EGFR could suppress the activation state of the pathway. Furthermore, in TRIM29-overexpressing chondrocytes, pharmacological inhibition with LY294002 (a PI3K inhibitor) or gefitinib (an EGFR inhibitor) reduced the expression of p-PI3K and p-AKT and autophagy activity (**Figs. 7G-H, S13**). Subsequently, SA-β-Gal staining revealed that TRIM29 overexpression increased senescence, but this effect was significantly alleviated by PI3K or EGFR inhibition; moreover, PI3K pathway blockade had a more pronounced rescue effect (**Fig. 7I-J**). Immunofluorescence analysis of P21 expression corroborated this trend (**Fig. 7K-L**). Additionally, SASP factors (IL-6, IL-10, MMP3, and MMP13) were upregulated upon TRIM29 overexpression and downregulated following inhibitor treatment (**Fig. 7M**). A rescue experiment was conducted to functionally validate the PI3K-AKT pathway as the key mediator downstream of TRIM29. While TRIM29 knockdown alleviated senescence, reactivation of PI3K-AKT signaling with AE18 effectively reversed this protective effect and restored a high level of senescence (**Fig. S14**). These data collectively demonstrate that TRIM29 directly targets PI3K subunits or indirectly engages EGFR to activate PI3K-AKT signaling, thereby inhibiting autophagy to promote chondrocyte senescence.

### 3.5 Delactylase SIRT1 inhibits OA progression

**Figure 8:**
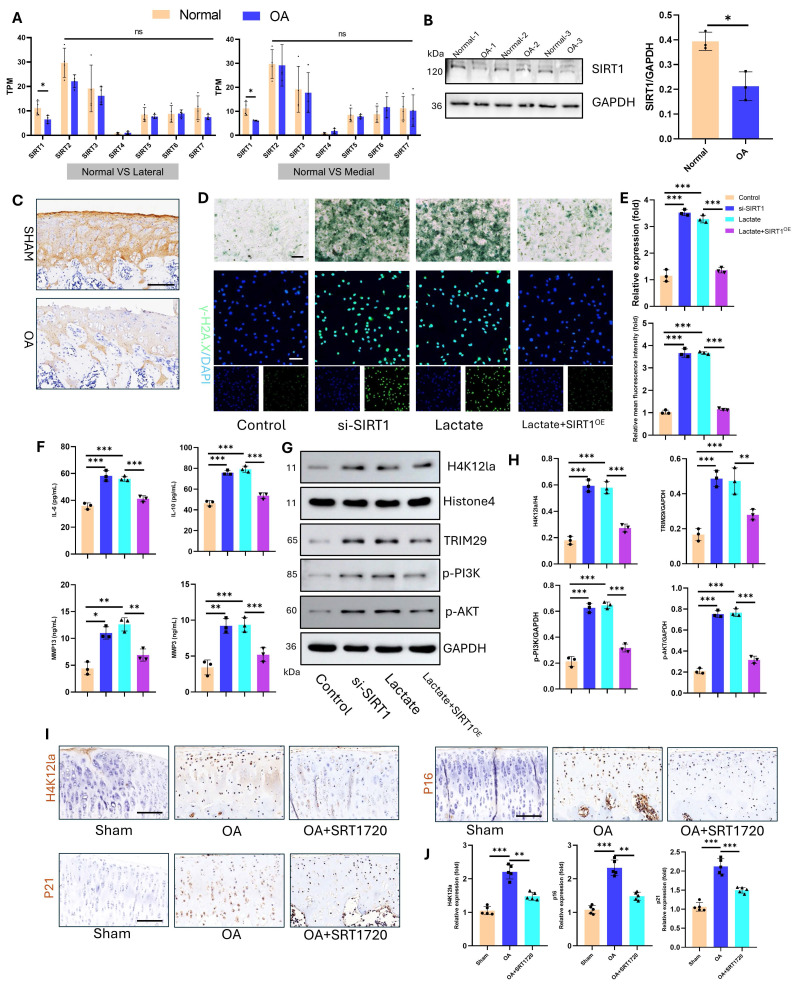
Overexpression of SIRT1 suppresses lactylation levels and chondrocyte senescence. A) Gene expression of sirtuin family proteins in articular cartilage from healthy donors and OA patients (including the lateral and medial compartments) (n=4). B) Western blot analysis of SIRT1 expression in articular cartilage (n=3). C) IHC detection of SIRT1 in articular cartilage from an SD rat OA model (n=5). Scale bar, 100 μm. D, E) SA-β-Gal activity and γ-H2AX expression were measured to evaluate cellular senescence (n=3). Scale bar, 100 μm. F) ELISA analysis of SASP components in chondrocytes to assess senescence (n=3). G, H) Western blotting analysis of H4K12la, TRIM29, p-PI3K, and p-AKT expression was performed to determine the delactylase activity of SIRT1 (n=3). I, J) IHC analysis of H4K12la, P16, and P21 expression to validate the effect of SIRT1 overexpression (via SRT1720 articular injection, 20 mg/kg) on lactylation and senescence in cartilage (n=5). Scale bar, 100 μm. *P < 0.05, **P < 0.01, ***P < 0.001.

The regulation of lactylation involves specific “writers” and “erasers.” Sirtuin 1-7 (SIRT1-7) enzymes are key delactylation erasers that modulate cellular activities^32^. Sirtuins—termed “longevity proteins”—also suppress cellular aging^33^. However, their role in osteoarthritis (OA) chondrocyte aging remains unclear. Transcriptomic analysis of human articular cartilage revealed the most pronounced change in the expression of SIRT1 compared with that of SIRT1-7 (**Fig. 8A**). Western blotting confirmed significantly reduced SIRT1 protein levels in OA cartilage (**Fig. 8B**). Consistent with this, IHC staining of an SD rat OA model demonstrated marked SIRT1 downregulation (**Fig. 8C**). Mechanistic studies in vitro examined SIRT1's effects on chondrocyte aging and lactylation under high lactate. After knockdown with siRNA and overexpression, we detected the expression of SIRT1 via a plasmid and quantified it via qPCR and Western blotting for subsequent functional assays (**Fig. S15**). SIRT1 knockdown (si-SIRT1) increased SA-β-Gal activity and γ-H2AX expression, whereas SIRT1 overexpression (SIRT^OE^) reversed the changes in these aging markers (**Fig. 8D-E**). ELISAs revealed that si-SIRT1 upregulated SASP factors (IL-6, IL-10, MMP3, and MMP13), whereas SIRT^OE^ attenuated SASP factor expression (**Fig. 8F**). SIRT1 directly regulates lactylation dynamics. SIRT1 knockdown increased H4K12la levels, whereas SIRT^OE^ reduced H4K12la, TRIM29, p-PI3K, and p-AKT expression under high-lactate conditions, confirming the delactylation function and downstream modulation of SIRT1 (**Fig. 8G-H**). For therapeutic validation in vivo, we used intra-articular SRT-1720 (a SIRT1 activator) in the OA joints of SD rats. IHC confirmed that SRT-1720 suppressed H4K12la, p16, and p21 levels, inhibiting both lactylation and chondrocyte aging (**Fig. 8I-J**). Thus, SIRT1 functions as a key eraser of lactylation in OA cartilage and a therapeutic target for mitigating OA-associated aging.

### 3.6 Suppressing glycolysis attenuates OA cartilage damage

**Figure 9:**
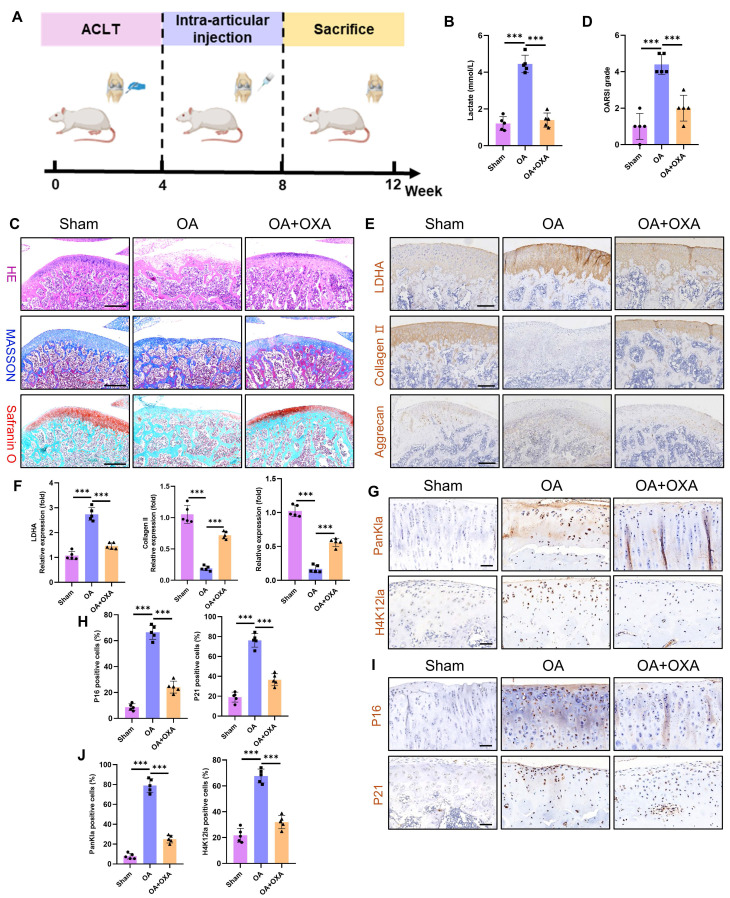
Glycolysis inhibition ameliorates cartilage damage during OA progression. A) Schematic of the animal experiment: four weeks after ACLT surgery, the SD rats received intra-articular injections of OXA (500 mg/kg) to inhibit glycolytic activity in the knee joint. B) Measurement of intra-articular lactate levels to evaluate the extent of glycolytic activity (n=5). C) Histopathological evaluation of articular cartilage via H&E, Masson and SO-FG staining to assess cartilage damage (n=5). Scale bar, 500 μm. D) OARSI scoring of knee articular cartilage in SD rats (n=5). E, F) IHC analysis of LDHA, collagen II, and aggrecan was performed to evaluate glycolytic activity and anabolic metabolism in cartilage (n=5). Scale bar, 200 μm. G, H) IHC detection of PanKla and H4K12la levels to assess lactylation activity in articular cartilage (n=5). Scale bar, 100 μm. I, J) IHC staining of P16 and P21 to determine the extent of chondrocyte senescence in articular cartilage (n=5). Scale bar, 100 μm. *P < 0.05, **P < 0.01, ***P < 0.001.

The primary therapeutic objective in OA is to prevent disease progression and ameliorate patient symptoms. Given that glycolytic hyperactivity drives chondrocyte senescence and cartilage degeneration via lactate accumulation, we investigated the therapeutic potential of the glycolysis inhibitor OXA to suppress intra-articular glycolysis in OA. Using an ACLT model in Sprague‒Dawley rats, we administered intra-articular OXA weekly from postoperative weeks 4-8. The tissues were harvested at 12 weeks for analysis (**Fig. 9A**). Cartilage lactate concentrations were markedly elevated in OA patients compared with those in sham controls, whereas OXA treatment significantly reduced lactate levels (**Fig. 9B**). Histopathological assessment (H&E, Masson's trichrome, and SO-FG staining) revealed severe cartilage erosion, structural disorganization, and subchondral bone calcification in the OA joints (**Fig. 9C**). OXA attenuated these degenerative changes, with OARSI scoring confirming significant mitigation of cartilage damage (**Fig. 9D**). Immunohistochemistry revealed upregulated LDHA (a glycolytic marker) and downregulated collagen II and aggrecan (anabolic markers) in OA cartilage (**Fig. 9E-F**). OXA reversed these effects, suppressing LDHA while restoring matrix protein expression. Interestingly, OXA also reduced PanKla and H4K12la lactylation modifications (**Fig. 9G-H**), implicating lactate-derived histone modifications in OA pathogenesis. Furthermore, OXA diminished chondrocyte senescence, as evidenced by reduced p16 and p21 expression (**Fig. 9I-J**). These results establish that glycolytic inhibition alleviates OA pathology by attenuating lactate-driven protein lactylation, chondrocyte senescence, and cartilage degradation. Our results establish that targeting chondrocyte glycolysis represents a promising metabolic strategy to decelerate OA progression, suggesting a novel therapeutic paradigm for this debilitating disease.

## 4. Discussion

This study presents the penetrating comprehensive analysis of how lactate derived from glycolysis drives chondrocyte senescence in OA and delineates the lactylation-dependent signaling pathway underlying this process. We showed that glycolytic activity is significantly increased in OA chondrocytes, resulting in markedly elevated lactate levels within the articular microenvironment. Further experiments demonstrated that lactate increases H4K12la, which activates the TRIM29/PI3K/AKT axis and promotes cellular senescence. Critically, both SIRT1 overexpression and pharmacological glycolysis inhibition attenuated lactate-induced lactylation in OA, thereby reducing cellular senescence and alleviating cartilage degradation. Together, these results provide mechanistic support for novel therapeutic approaches targeting metabolic reprogramming and lactylation-driven senescence in osteoarthritic chondrocytes.

While OA was historically characterized as low-grade chronic inflammation of the whole joint, recent evidence redefines it as a metabolic disorder^10^, ^11^, ^34^. Central to this paradigm shift is the dysregulation of articular cartilage energy metabolism. Physiologically, the avascular nature of cartilage restricts oxygen tension to 1-6%, which is significantly lower than the 13% oxygen tension at arterial levels^12^; thus, chondrocytes rely primarily on glycolysis for energy production^35^, ^36^. Crucially, OA progression coincides with marked upregulation of glycolytic rate-limiting enzymes in chondrocytes, increasing glycolytic flux and lactate accumulation^8^, ^9^, ^37^, ^38^. This metabolic reprogramming is clinically significant: bioinformatics and machine learning approaches have established glycolytic signatures as diagnostic biomarkers and therapeutic targets for OA^39^. Our findings align with and extend this evidence. Transcriptomic analysis of OA patient cartilage revealed significant enrichment in glycolysis-related pathways (glycolysis/gluconeogenesis, HIF-1 signaling, and carbon metabolism) and elevated expression of glycolytic rate-limiting enzymes. IL-1β stimulation elicited a pro-glycolytic shift (increased PKM2/LDHA), elevated lactate, and reduced ATP in chondrocytes and OA model rats, culminating in accelerated cartilage degradation, thereby corroborating the human findings. These results demonstrate that glycolytic metabolic reprogramming in chondrocytes directly regulates OA pathogenesis.

Historically, lactate has been widely regarded as a metabolic waste product of glycolysis. However, accumulating evidence establishes lactate as both a crucial metabolic carbon source and a key signaling molecule in cellular communication, particularly within tumors and chronic inflammatory microenvironments^40^, ^41^. In OA articular cartilage, increased glycolytic activity leads to substantial accumulation of lactate. This elevated lactate concentration enhances catabolism of the extracellular matrix in chondrocytes, thereby contributing to OA progression^42^. Nevertheless, the precise mechanisms through which lactate exacerbate OA remain incompletely understood. In this study, we demonstrated that treatment with IL-1β and exogenous lactate intensified senescence-associated activities in chondrocytes. Conversely, when glycolytic flux was inhibited with OXA, IL-1β-induced cellular senescence was attenuated. These findings indicate that IL-1β promotes glycolytic activity in chondrocytes, leading to increased lactate production, which in turn creates a prosenescent microenvironment.

Lactylation is an emerging post-translational modification that utilizes lactate as a substrate to modify target proteins. This process, integral to metabolic reprogramming, exerts broad regulatory functions spanning multiple disease contexts^43^-^45^. In OA, lactate has traditionally been viewed as a metabolic byproduct and disease biomarker; however, its role in epigenetic regulation via lactylation remains poorly explored. Previous studies have indicated that UGDH lactylation at K6 disrupts its interaction with STAT1, activating the MAPK pathway and promoting ECM degradation and apoptosis^46^. Similarly, elevated H3K18la levels in OA chondrocytes contribute to cellular damage and disease progression^47^, ^48^. Consistent with these findings, our study revealed a marked upregulation of PanKla in OA cartilage. Furthermore, we identified a specific increase in H4K12la, a histone modification site previously uncharacterized in OA, through experimental validation. The functional significance of H4K12la in OA, however, remains unclear. To investigate its downstream regulatory role, we performed H4K12la-specific CUT&TAG sequencing on articular cartilage from healthy donors and OA patients. This analysis revealed that TRIM29 is a key potential target of H4K12la-mediated regulation. Together, luciferase and ChIP-qPCR analyses demonstrated significant enrichment of the H4K12la mark at the TRIM29 promoter.

TRIM29, a member of the TRIM family that uniquely lacks a RING-finger domain^49^, has been implicated in cancer, diabetic nephropathy, and immune-related diseases and is considered a promising diagnostic marker and therapeutic target^50^. However, its role in OA chondrocyte senescence has not been previously examined. Here, we demonstrated that both IL-1β and exogenous lactate upregulate TRIM29 expression in chondrocytes. Elevated TRIM29 levels exacerbated cellular senescence, whereas TRIM29 knockdown significantly attenuated lactate-induced senescence. These results indicate that lactate-induced H4K12la promotes TRIM29 expression, thereby accelerating chondrocyte senescence. This finding aligns with growing evidence linking lactylation to senescence regulation across pathological contexts^17^, ^51^, ^52^. We previously showed that lactate activates the PI3K-AKT pathway to induce senescence. Intriguingly, TRIM29 has been reported to interact closely with PI3K-AKT signaling, although its effects appear context dependent. In colorectal cancer, TRIM29 ubiquitinates and degrades PHLPP1, leading to PI3K-AKT activation and increased proliferation, migration, and invasion^53^. Conversely, in liver cancer, TRIM29 promotes YBX1 degradation to suppress PI3K-AKT signaling and reverse drug resistance^49^. In our study, we found that TRIM29 directly binds subunits of PI3K, activating downstream signaling, and facilitates EGFR binding, resulting in dual activation of the PI3K-AKT pathway, which promotes chondrocyte senescence. On the basis of these findings, we propose a novel regulatory axis in OA: lactate accumulation drives H4K12la upregulation, which enhances TRIM29 expression. TRIM29, in turn, dually activates PI3K-AKT signaling through direct and EGFR-mediated mechanisms, culminating in accelerated chondrocyte senescence and OA progression. To our knowledge, this is the first study to delineate a lactylation-dependent mechanism regulating senescence in OA chondrocytes, providing new insights into OA pathogenesis and highlighting potential therapeutic targets.

The relationship between lactate and cellular senescence is complex and context dependent and varies across pathological conditions. For example, in vascular smooth muscle cells, TRAP1-mediated metabolic reprogramming enhances aerobic glycolysis and lactate production. Subsequent lactate accumulation triggers H4K12 lactylation, which in turn activates SASP transcription. This process aggravates VSMC senescence and SASP gene expression, thus accelerating cellular senescence and promoting atherosclerosis^52^. In contrast, lactate exposure in lung cancer cells upregulates Snail protein expression via ECM remodeling and TGF-β1 activation, suppresses p16 transcription, and facilitates escape from senescence^15^. In OA, chondrocyte senescence is a well-established hallmark of the disease, and senolytic interventions are emerging as promising therapeutic strategies^6^, ^54^. However, the regulatory influence of lactylation on chondrocyte senescence in OA has not been elucidated. Here, we revealed that lactate-induced lactylation activates the PI3K-AKT signaling pathway, which suppresses autophagy and promotes senescence. The PI3K-AKT pathway is recognized as an upstream negative regulator of autophagy^55^. Autophagic activity in chondrocytes is essential for clearing damaged cytoplasmic components and modulating inflammatory responses. However, impaired autophagic flux during OA progression disrupts intracellular homeostasis, leading to increased chondrocyte senescence and death, along with loss of cartilage integrity^56^, ^57^. In summary, our results provide compelling evidence that lactate-induced lactylation accumulation activates PI3K-AKT signaling, inhibits protective autophagy, aggravates chondrocyte senescence, and ultimately accelerates OA pathogenesis.

The regulation of protein lactylation is mediated by specific enzymatic writers and erasers. SIRT1 has been identified as a key eraser of lactylation^32^. Previous studies have demonstrated that SIRT1 overexpression suppresses lactate-induced lactylation, thereby conferring therapeutic benefits in various disease models^58^, ^59^. Furthermore, SIRT1 activation counteracts stem cell aging and promotes aged bone regeneration, consistent with its role as a longevity factor 33, 60. Consistent with earlier reports confirming SIRT1 downregulation in OA^61^, our transcriptomic analysis revealed that SIRT1 is the only member of the sirtuin family that is significantly reduced in OA cartilage. Targeted activation or delivery of SIRT1 into chondrocytes has been shown to mitigate cartilage degradation and cellular senescence^62^, ^63^. In line with these findings, we observed that SIRT1 overexpression in chondrocytes significantly reduced H4K12la levels, leading to inhibition of the TRIM29-PI3K-AKT signaling pathway and attenuation of cellular senescence. Similarly, intra-articular injection of a SIRT1 agonist in a rat OA model markedly decreased lactylation levels and senescence activity in articular cartilage, thereby ameliorating cartilage damage.

Although this study elucidates the mechanism by which lactate-induced lactylation regulates chondrocyte senescence in OA, several limitations remain. First, we did not collect comprehensive clinical data—such as age, sex, and biochemical profiles—from OA patients to correlate lactylation levels with senescence markers in vivo. Additionally, the effects of lactylation on other chondrocyte processes, including autophagy, apoptosis, and ferroptosis, remain unexplored. Although in vivo intervention experiments support the existence of the entire pathway, developing tools for specific knockout of TRIM29 or intervention of H4K12la in cartilage will ultimately be able to verify the causal relationship of this axis in vivo. These aspects warrant further investigation in subsequent studies.

## 5. Conclusion

In summary, this study revealed that increased glycolytic flux in OA articular cartilage and lactate-induced protein lactylation collectively promote chondrocyte senescence and subsequent cartilage degeneration. Mechanistically, lactate upregulates H4K12la, which enhances the transcription of TRIM29. TRIM29, in turn, activates the PI3K-AKT signaling pathway through both direct interaction and EGFR-mediated mechanisms, promoting chondrocyte senescence. Importantly, the inhibition of lactylation or glycolytic activity markedly attenuates cartilage damage and mitigates OA progression. The metabolic reprogramming-cell senescence axis identified here establishes a novel conceptual framework for OA pathogenesis and offers a robust theoretical foundation for the innovative development of targeted therapeutics.

## Supplementary Material

Supplementary figures and tables.

## Data Availability

The datasets generated and/or analyzed during the current study are available from the corresponding author on reasonable request.

## Abbreviations

OA: osteoarthritis
SASP: senescence-associated secretory phenotype
ACLR: anterior cruciate ligament reconstruction
TKA: total knee arthroplasty
TPM: transcripts per million
ACLT: anterior cruciate ligament transaction
SD: sprague Dawley
PBS: phosphate-buffered saline
qRT-PCR: quantitative reverse transcription polymerase chain reaction
RT: room temperature
ECAR: extracellular acidification rate
2-DG: 2-deoxyglucose
H&E: hematoxylin and eosin
SO-FG: safranin o-fast green
IHC: immunohistochemistry
CCK-8: cell counting kit-8
SA-β-Gal: senescence-associated β-galactosidase
ChIP: Chromatin Immunoprecipitation
ELISA: enzyme-linked immunosorbent assay
Co-IP: co-immunoprecipitation
ANOVA: analysis of variance
DEGs: differentially expressed genes
OXA: oxamate
TRIM29: tripartite motif-containing protein 29
PanKla: panlysine lactylation
IGV: integrative genomics viewer
