# Supplementary material for: Investigating the Mechanistic Link Between Lactate-Induced Histone Lactylation and Cellular Senescence in Osteoarthritis Chondrocytes: Implications for Therapy
Source: Int J Biol Sci. 2026 Feb 18;22(6):2925–49. doi: 10.7150/ijbs.126483 (PMC13050444; doi:10.7150/ijbs.126483)
Supplement: Supplementary file 1 — Supplementary figures and tables. [file ijbsv22p2925s1.pdf]

Supplementary Materials

Investigating the Mechanistic Link Between Lactate-Induced Histone Lactylation and Cellular Senescence in Osteoarthritis Chondrocytes: Implications for Therapy

Table S1 Information of healthy and OA patients used in study.

**NO.**	**Gender**	**Age**	**NO.**	**Gender**	**Age**	**Grade**
Healthy-1	Male	18	OA-1	Female	69	IV
Healthy-2	Female	26	OA-2	Female	70	IV
Healthy-3	Female	27	OA-3	Female	76	IV
Healthy-4	Male	22	OA-4	Female	73	IV

Table S2 Sequences of Primers used for qPCR

Genes	Forward primer sequence (5′–3′)	Reverse primer sequence (5′–3′)
Collagen II	CTCATCCAGGGCTCCAATGA	CCATGGGTGCAATGTCAACA
Aggrecan	CAGTGCGATGCAGGCTGGCT	CCTCCGGCACTCGTTGGCTG
SOX9	GGATGTCAAAGCAACAGGCG	ATGTGCGTTCTCTGGGACTG
MMP3	GCTGTTTTTGAAGAATTTGGGTTC	GCACAGGCAGGAGAAAACGA
MMP13	GTGACAGGAGCTAAGGCAGA	AGCATGAAAGGGTGGTCTCA
TRIM29	CTCCCTGAAAGGCTATCCCTC	TGGCCGGTAGTGAGACAGTA
SIRT1	TATGCTCGCCTTGCTGTG	CAGAGATGGCTGGAACTGTC

Table S3 Primary antibodies used for western blotting, IF and IHC

Name	Corporation	kDa	Application	Item Number
GLUT1	Proteintech	50	WB	21829-1-AP
PKM2	Proteintech	58	WB/IF	15822-1-AP
LDHA	Proteintech	35	WB/IF	19987-1-AP
HK2	Proteintech	102	WB	22029-1-AP

Ki67	Proteintech	-	IF	27309-1-AP
P16	Proteintech	-	IHC	10883-1-AP
P21	ABclonal	43	IF/IHC	A1483
γ-H2AX	Proteintech	66-70	IF	68888-1-Ig
Pan Kla	PTM Bio	-	WB/IF	PTM-1401RM
H3K19la	PTM Bio	15	WB	PTM-1419RM
H3K14la	PTM Bio	15	WB	PTM-1414RM
H4K8la	PTM Bio	11	WB	PTM-1415RM
H4K12la	PTM Bio	11	WB/IF	PTM-1411RM
H4K16la	PTM Bio	11	WB	PTM-1417RM
H3	PTM Bio	15	WB	PTM-1001RM
H4	PTM Bio	11	WB	PTM-1015RM
TRIM29	Proteintech	65	WB/CO-IP	17542-1-AP
SIRT1	Proteintech	120	WB	13161-1-AP
PI3K p110α	Proteintech	110	WB/CO-IP	67071-1-Ig
PI3K p110β	Proteintech	110	WB/CO-IP	20584-1-AP
PI3K p85α	Proteintech	85	WB/CO-IP	60225-1-Ig
PI3K p85β	Proteintech	85	WB/CO-IP	67644-1-Ig
EGFR	Proteintech	134	WB/CO-IP	18986-1-AP
p-PI3K	MCE	85	WB	HY-P80846
p-AKT	Abcam	60	WB	ab81283
LC3	Abcam	14,16	WB	ab63817
P62	Abcam	62	WB	ab109012
ATG5	Abcam	50	WB	ab108327
Beclin1	Abcam	52	WB	ab207612
Collagen II	Proteintech	-	IHC	28459-1-AP
Aggrecan	ABclonal	-	IHC	A8536
GAPDH	Proteintech	36	WB	60004-1-Ig

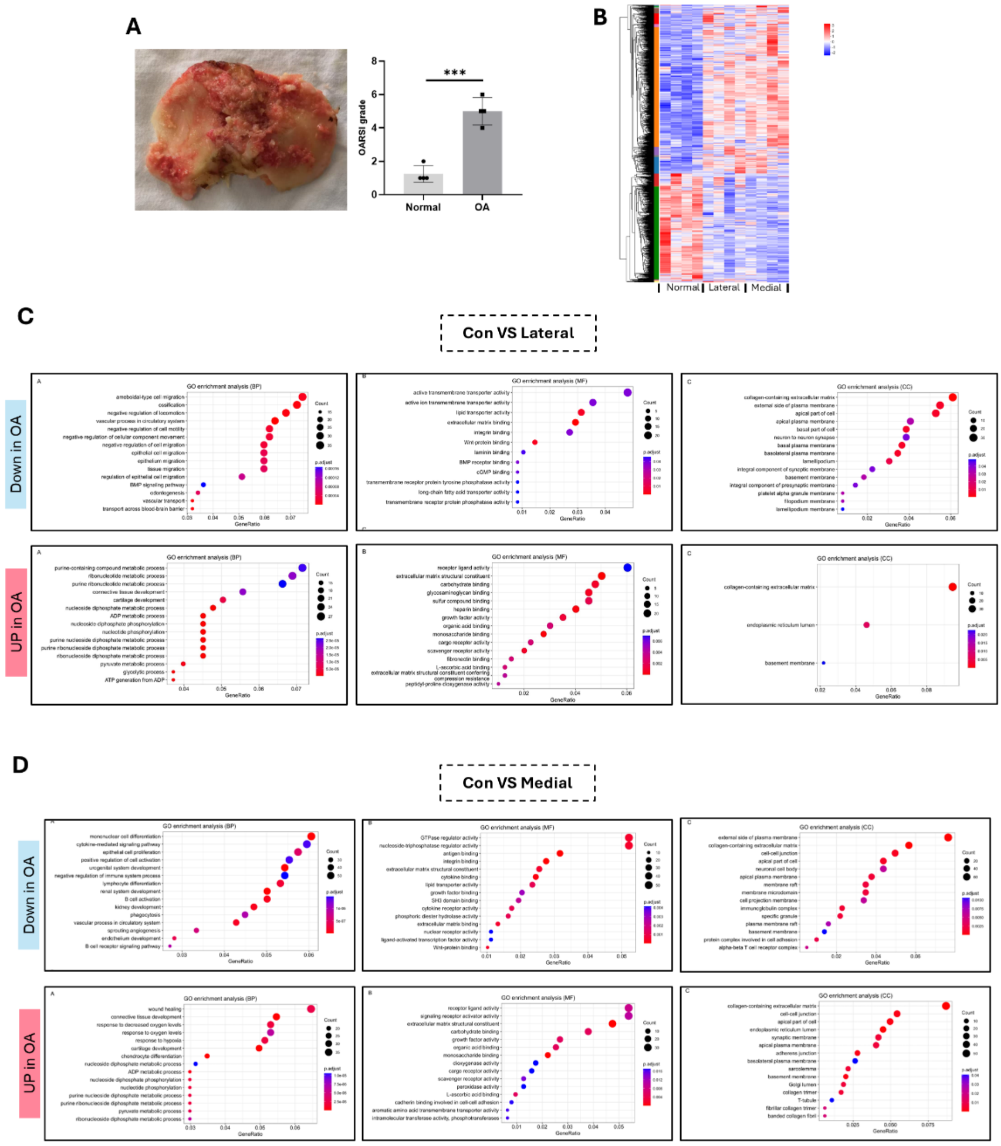

Fig S1 A) Gross appearance of the tibial plateau and OARSI score of articular cartilage from OA patients following TKA. B) Heatmap of transcriptomic sequencing analysis of articular cartilage. C, D) GO enrichment analysis of up-regulated and down-regulated genes in OA articular cartilage (including lateral and medial compartments). ***$P < 0.001$.

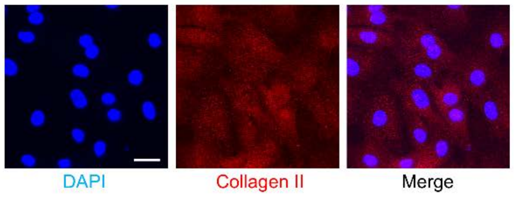

Fig S2 The expression of Collagen II in the extracted SD rat chondrocytes.

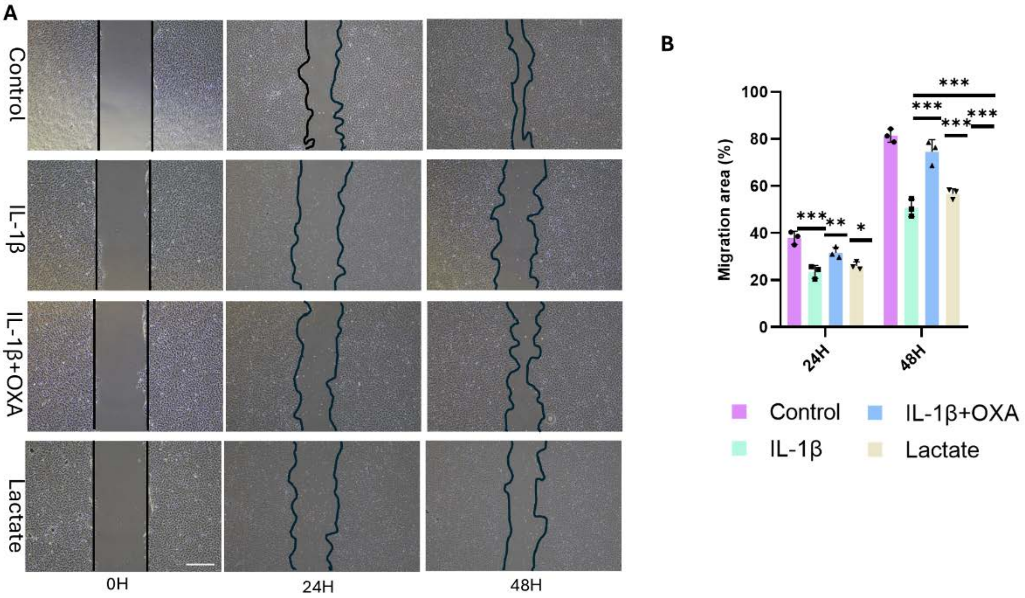

Fig S3 A-B) Scratch assay was used to determine the migration ability of chondrocytes. *$P < 0.05$, **$P < 0.01$, ***$P < 0.001$.

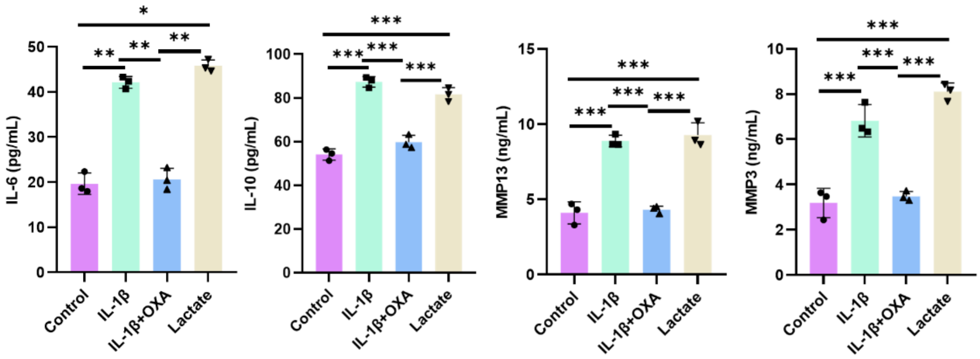

Fig S4 ELISA detection for the SASP components. *$P < 0.05$, **$P < 0.01$, ***$P < 0.001$.

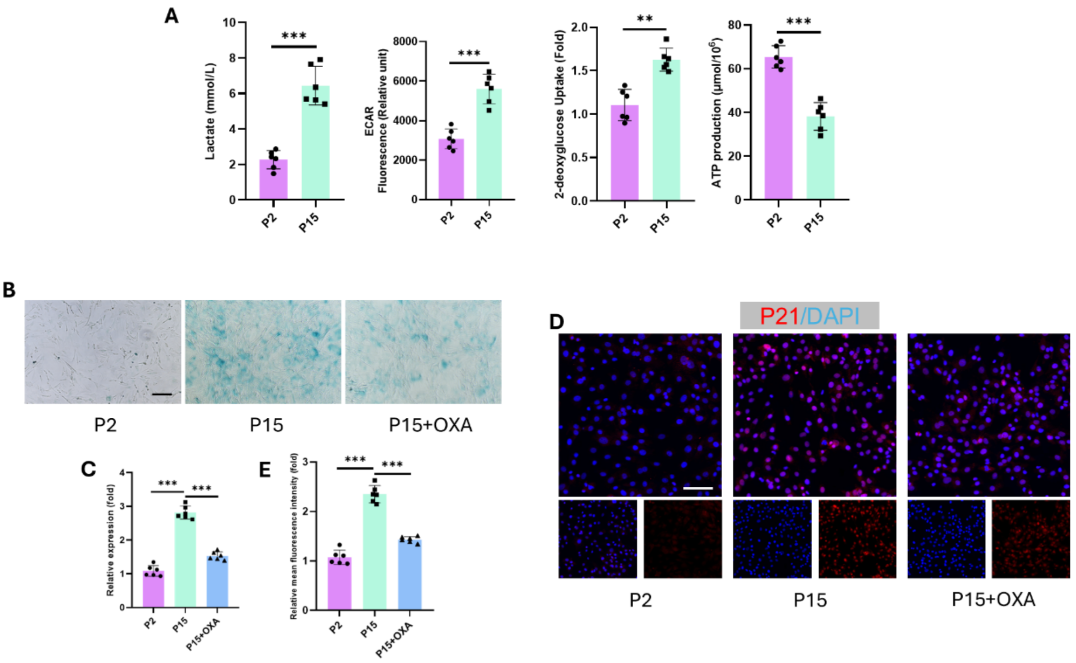

Fig S5 Glycolytic activity is enhanced in naturally aged chondrocytes. A) Chondrocytes at passage 15 (P15) exhibit significantly enhanced glycolytic activity compared to those at passage 2 (P2), as indicated by increased lactate production, elevated ECAR, enhanced glucose uptake, and reduced ATP production. B, C) SA-β-gal staining reveals increased senescence in P15 chondrocytes, which is markedly attenuated following inhibition of glycolysis with OXA. Scale bar, 100 μm. D, E) Immunofluorescence confirms elevated P21 expression in P15 chondrocytes, which is significantly reduced upon OXA-mediated glycolytic inhibition. Scale bar, 100 μm. $^{**}P < 0.01$, $^{***}P<0.001$.

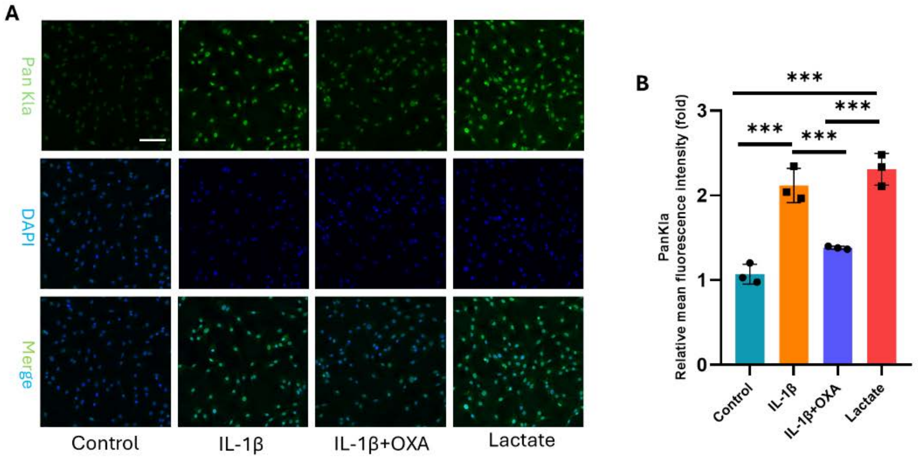

Fig S6 A-B) Immunofluorescence staining confirms elevated PanKla expression in chondrocytes following treatment with IL-1β and lactate. Scale bar, 100 μm. $^{***}P < 0.001$.

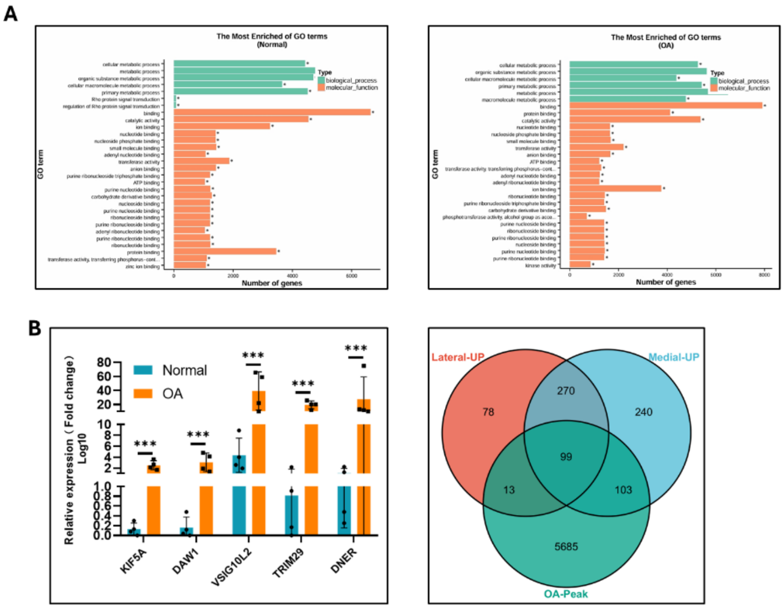

Fig S7 A) GO enrichment analysis of genes associated with H4K12la peaks from CUT&TAG sequencing in healthy and OA groups. **B)** Expression levels of KIF5A, DAW1, VISG10L2, TRIM29, and DNER in articular cartilage from healthy donors and OA patients. Integration of transcriptomic sequencing and CUT&TAG peak genes in OA was performed to identify H4K12la target genes.

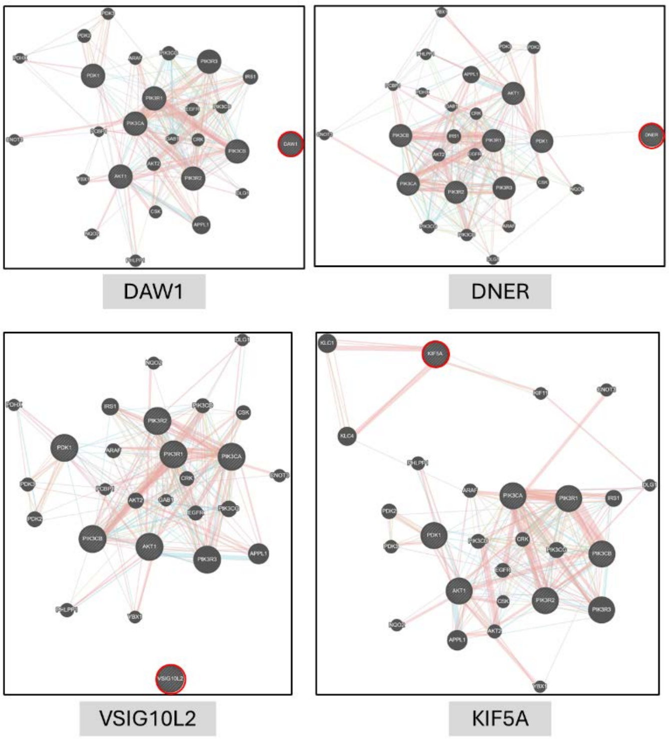

Fig S8 GeneMANIA analysis predicting potential functional interactions between DAW1, DNER, VSIG10L2, KIF5A and the PI3K pathway.

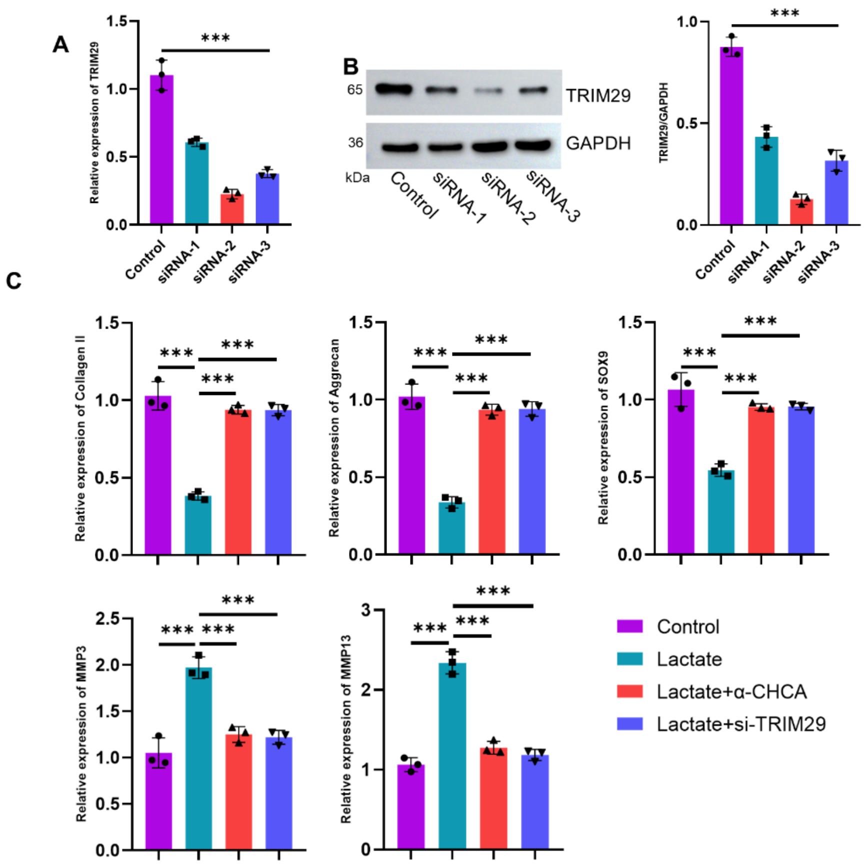

Fig S9 A, B) Efficiency of TRIM29 knockdown by siRNA-TRIM29 was measured using qPCR and western blotting analysis. C) Effect of lactate on the expression of chondrocyte anabolic markers (collagen II, aggrecan, and SOX9) and catabolic markers (MMP3 and MMP13) following TRIM29 knockdown, as detected by qPCR. ***$P < 0.001$.

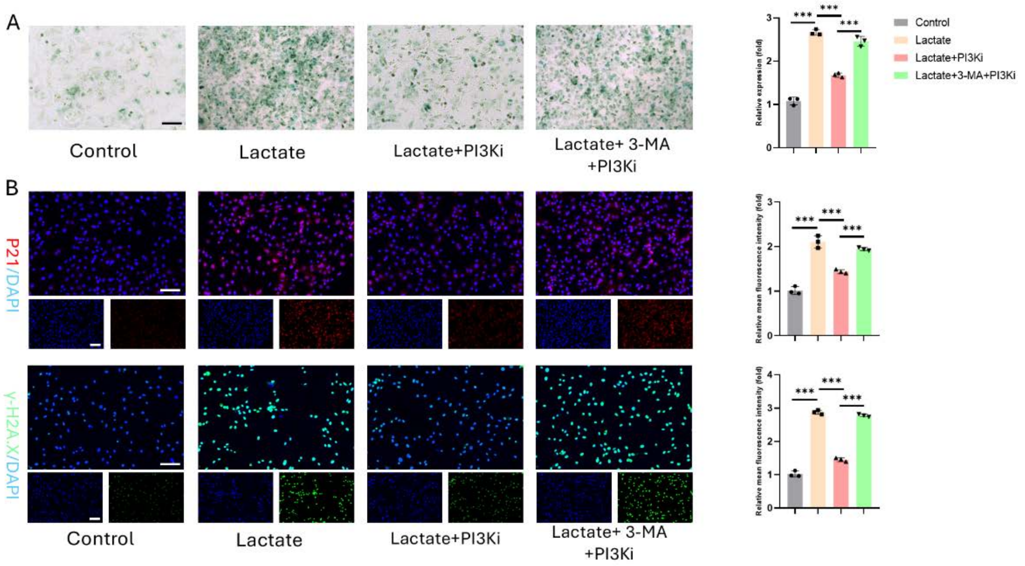

Fig S10 A, B) Lactate exacerbates cellular senescence through PI3K-mediated suppression of

autophagy, leading to increased SA-β-gal activity and elevated expression of P21 and γ-H2AX. **P* < 0.001.

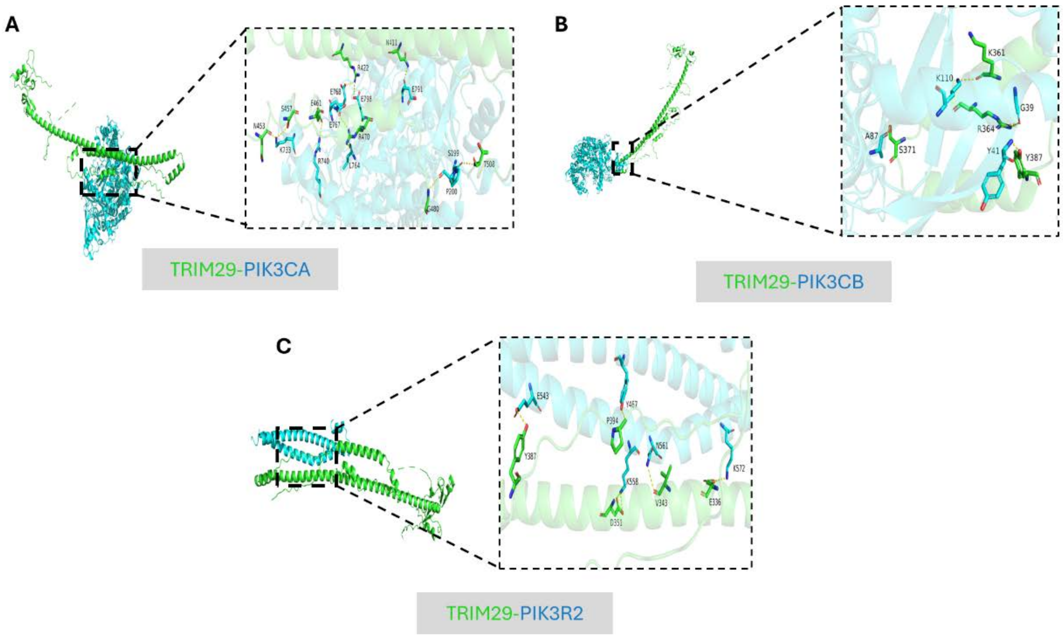

Fig S11 A–C) Schematic representation of molecular docking simulations between TRIM29 and PIK3CA, PIK3CB, and PIK3R2.

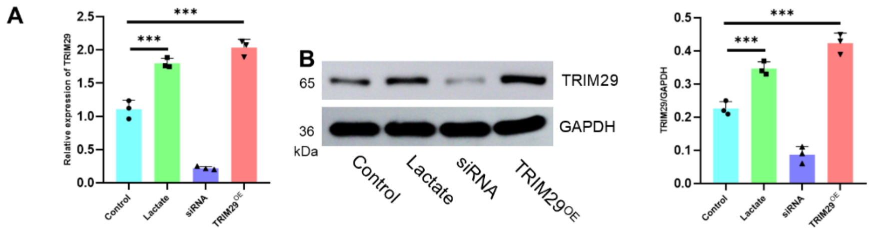

Fig S12 A, B) The expression of TRIM29 after transfection was detected by qPCR and western blotting. ****P* < 0.001.

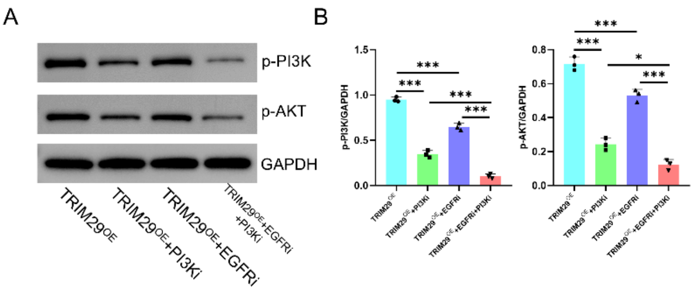

Fig S13 A, B) In chondrocytes overexpressing TRIM29, pharmacological inhibitors reduce the protein levels of both PI3K and EGFR. **P* < 0.05, ****P* < 0.001.

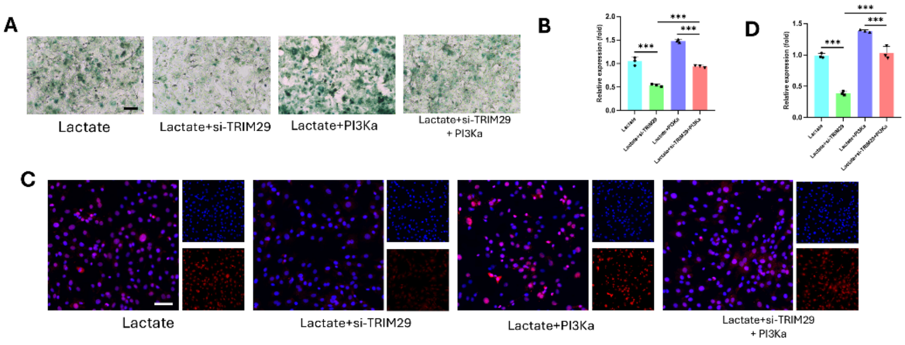

Fig S14 A, B) The rescue experiment aims to functionally verify that the PI3K-AKT pathway is the key transmission pathway through which TRIM29 regulates cellular aging activities. ***$P < 0.001$.

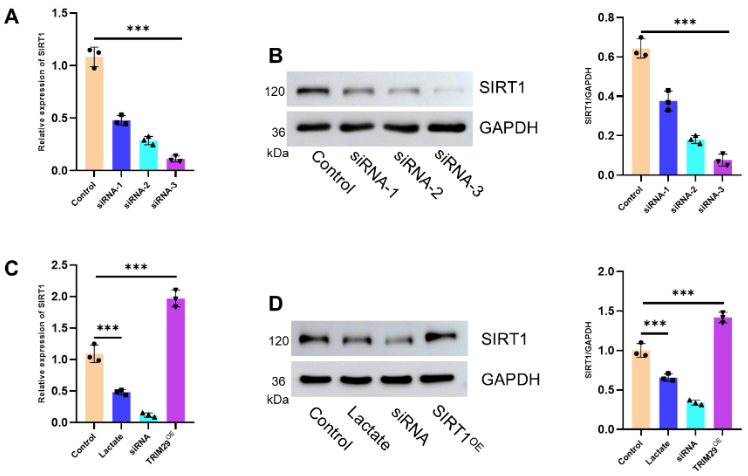

Fig S15 A, B) Efficiency of SIRT1 knockdown by siRNA was measured using qPCR and western blotting analysis. C, D) The expression of TRIM29 after transfection was detected by qPCR and western blotting. ***$P < 0.001$.
